# Global impacts of the 1980s regime shift

**DOI:** 10.1111/gcb.13106

**Published:** 2015-11-23

**Authors:** Philip C. Reid, Renata E. Hari, Grégory Beaugrand, David M. Livingstone, Christoph Marty, Dietmar Straile, Jonathan Barichivich, Eric Goberville, Rita Adrian, Yasuyuki Aono, Ross Brown, James Foster, Pavel Groisman, Pierre Hélaouët, Huang‐Hsiung Hsu, Richard Kirby, Jeff Knight, Alexandra Kraberg, Jianping Li, Tzu‐Ting Lo, Ranga B. Myneni, Ryan P. North, J. Alan Pounds, Tim Sparks, René Stübi, Yongjun Tian, Karen H. Wiltshire, Dong Xiao, Zaichun Zhu

**Affiliations:** ^1^ Sir Alister Hardy Foundation for Ocean ScienceThe LaboratoryCitadel HillPlymouthPL1 2PBUK; ^2^ Marine InstitutePlymouth UniversityDrake CircusPlymouthPL4 8AAUK; ^3^ Marine Biological Association of the UKThe LaboratoryCitadel HillPlymouthPL1 2PBUK; ^4^ EawagSwiss Federal Institute of Aquatic Science and TechnologyÜberlandstrasse 133CH‐8600DübendorfSwitzerland; ^5^ Centre National de la Recherche ScientifiqueLaboratoire d'Océanologie et de Géosciences (LOG)UMR 8187 LOGUniversité des Sciences et Technologies de LilleBP 8062930WimereuxFrance; ^6^ WSL Institute for Snow and Avalanche Research SLFFlüelastrasse 11CH‐7260DavosSwitzerland; ^7^ Department of BiologyLimnological InstituteUniversity of Konstanz78464KonstanzGermany; ^8^ Climatic Research UnitSchool of Environmental SciencesUniversity of East AngliaNorwichNR4 7TJUK; ^9^ Laboratoire des Sciences du Climat et de l'EnvironnementCEA‐CNRS‐UVSQL'Orme des Merisiers91191Gif‐sur‐YvetteFrance; ^10^ Department of Ecosystem ResearchLeibniz‐ Institute of Freshwater Ecology and Inland FisheriesMüggelseedamm 301D‐12587BerlinGermany; ^11^ Graduate School of Life and Environmental SciencesOsaka Prefecture UniversitySakai599‐8531Japan; ^12^ Climate Research DivisionScience and Technology BranchEnvironment Canada Ouranos550 Sherbrooke St. West19th FloorMontréalQCH3A 1B9Canada; ^13^ Code 917NASA/Goddard Space Flight CenterGreenbeltMD20771USA; ^14^ National Centers for Environment Information ‐ Center for Weather and ClimateFederal Building151 Patton AvenueAshevilleNC28801USA; ^15^ P.P. Shirshov Institute for OceanologyRAS36 Nakhimovsky Avenue117997MoscowRussia; ^16^ Research Center for Environmental ChangesAcademia Sinica128 Academia Road, Section 2, NankangTaipei115Taiwan; ^17^ Met OfficeHadley CentreFitzRoy RoadExeterDevonEX1 3PBUK; ^18^ Alfred‐Wegener Institute for Polar and Marine ResearchBiologische Anstalt HelgolandKurpromenade 20127498HelgolandGermany; ^19^ College of Global Change and Earth System Science (GCESS)Beijing Normal UniversityBeijing100875China; ^20^ Joint Center for Global Change StudiesBeijing100875China; ^21^ Weather Forecast CenterCentral Weather Bureau64 Gongyuan RoadTaipei10048Taiwan; ^22^ Department of Earth and EnvironmentBoston University685 Commonwealth AvenueBostonMA02215USA; ^23^ Helmholtz‐Zentrum GeesthachtInstitute of Coastal ResearchMax‐Planck‐Str. 1D‐21502GeesthachtGermany; ^24^ Monteverde Cloud Forest PreserveTropical Science CenterSanta ElenaPuntarenas5655‐73Costa Rica; ^25^ Institute of ZoologyPoznań University of Life SciencesWojska Polskiego 71 C60‐625PoznańPoland; ^26^ Faculty of Engineering, Environment and ComputingCoventry UniversityCoventryCV1 5FBUK; ^27^ Fachgebiet für ÖkoklimatologieTechnische Universität MünchenHans‐Carl‐von‐Carlowitz‐Platz 285354FreisingGermany; ^28^ Institute for Advanced StudyTechnische Universität MünchenLichtenbergstrasse 2a85748GarchingGermany; ^29^ Federal Office of Meteorology and ClimatologyMeteoSwissCh. de l'Aérologie 1CH‐1530PayerneSwitzerland; ^30^ Fisheries CollegeOcean University of ChinaYushan Road 5Qingdao266003China; ^31^ Japan Sea National Fisheries Research InstituteFisheries Research AgencyChuo‐kuNiigata951‐8121Japan; ^32^ Chinese Academy of Meteorological SciencesBeijing100081China; ^33^ State Key Laboratory of Remote Sensing ScienceInstitute of Remote Sensing and Digital EarthChinese Academy of SciencesBeijing100101China; ^34^ Center for Applications of Spatial Information Technologies in Public HealthBeijing100101China

**Keywords:** climate, Earth systems, global change, regime shift, statistical analysis, time series, volcanic forcing

## Abstract

Despite evidence from a number of Earth systems that abrupt temporal changes known as regime shifts are important, their nature, scale and mechanisms remain poorly documented and understood. Applying principal component analysis, change‐point analysis and a sequential *t*‐test analysis of regime shifts to 72 time series, we confirm that the 1980s regime shift represented a major change in the Earth's biophysical systems from the upper atmosphere to the depths of the ocean and from the Arctic to the Antarctic, and occurred at slightly different times around the world. Using historical climate model simulations from the Coupled Model Intercomparison Project Phase 5 (CMIP5) and statistical modelling of historical temperatures, we then demonstrate that this event was triggered by rapid global warming from anthropogenic plus natural forcing, the latter associated with the recovery from the El Chichón volcanic eruption. The shift in temperature that occurred at this time is hypothesized as the main forcing for a cascade of abrupt environmental changes. Within the context of the last century or more, the 1980s event was unique in terms of its global scope and scale; our observed consequences imply that if unavoidable natural events such as major volcanic eruptions interact with anthropogenic warming unforeseen multiplier effects may occur.

## Introduction

Regime shifts are abrupt, substantial and persistent changes in the state of natural systems. Such shifts have been observed in the atmosphere (Lo & Hsu, 2010; Xiao *et al*., 2012), ecosystems (Hastings & Wysham, 2010) and human social systems (Campbell & Allen, 2001). Three regime shifts (1970s, 1980s and 1990s), distinguished by marked increases in temperatures or by abrupt temporal changes across different biophysical systems, have been identified in the last few decades (Hare & Mantua, 2000; Reid *et al*., 2001; Gong & Ho, 2002; Yasunaka & Hanawa, 2002; Peterson & Schwing, 2003; Beaugrand *et al*., 2013). Documented until now at ocean basin or regional scales, the mechanisms behind these events, their environmental interactions, and the synchrony and scale of their effects around the globe are poorly understood. There is thus a considerable research gap with many disparate observations by different scientific disciplines, but no comprehensive overall assessment. Here, we address this gap by focusing on the 1980s regime shift and show, using three independent statistical methods that this shift took place on a planetary scale and involved the carbon cycle (Beaulieu *et al*., 2012b); disease (Vezzulli *et al*., 2012); and biotic, physical and chemical components of land (Myneni *et al*., 1997; Brandt *et al*., 2013), freshwater (Hari *et al*., 2006), precipitation (Tao *et al*., 2015), marine (Möllmann & Diekmann, 2012; Beaugrand *et al*., 2015) as well as cryospheric (Brown & Robinson, 2011) and atmospheric (Lo & Hsu, 2010; Xiao *et al*., 2012) Earth systems. A total of 72 time series was processed and analysed statistically to represent as many natural systems as possible, and to illustrate shiftlike abrupt changes in a ‘1980s period of interest’ (1983 to 1990) between the volcanic eruptions of El Chichón and Pinatubo (see Materials and methods).

To explore possible mechanisms behind the 1980s regime shift, we used historical climate model simulations from the Coupled Model Intercomparison Project Phase 5 (CMIP5) (Jones *et al*., 2013) together with statistical modelling (Folland *et al*., 2013) of the main anthropogenic radiative forcing and natural (volcanic and solar) factors influencing global mean surface temperature. Using these approaches, we show that the rapid cooling of the Earth's surface (Robock, 2000), and especially of the oceans (Church *et al*., 2005), initiated by the El Chichón volcanic eruption of 1982 was followed by a recovery reinforced by anthropogenic warming. It is the scale and speed of these combined heating effects that we propose contributes to the synchronization of the regime shift between different systems. Although temperature appears to be the main forcing factor, volcanic and anthropogenic aerosols and their interactions with clouds (IPCC Chap. 7, 2013) and the brightening effect described by Wild (2009) may also have contributed. The 1980s regime shift is an example of unforeseen compounding effects that may occur if unavoidable natural events such as major volcanic eruptions interact with anthropogenic warming.

## Materials and methods

### Data

#### Time series selection

Long time series of variables representing the various key components of the climate system (drivers) and a wide range of environmental and ecological indicators (responders) were used for our study. Half the 72 time series represent global and hemispheric (26) to local (10) land, sea and freshwater temperatures, with the other half covering, at global to local scales, the carbon cycle (3) and the following natural systems: atmosphere (9), cryosphere (6), marine hydrosphere (2), marine biosphere (3), terrestrial hydrosphere (3) and terrestrial biosphere (10). We have used a variety of different forms of measurements with one value per year: for example, averaged over single months, seasons or yearly, or in the case of phenology the timing date. Some of the time series presented are averages of data sets where our analyses showed the shift in most of the members, for example 18 river water temperatures averaged for Switzerland or many gridded temperature data sets; others are representatives of vertical profiles in the atmosphere or ocean that show similar shifts at other different heights or depths. The majority of the time series are from the Northern Hemisphere. Few unbroken long‐term time series from the tropics exist that have been produced using the same standard protocols over time. Furthermore, time series data, other than for land surface temperature (LST) and sea surface temperature (SST) from ocean areas outside the North Atlantic and North Pacific, are very sparse.

Considerable effort was put into the search for long‐term time series and included the following: submissions from recommendations by the authors, downloading from open‐access online databases, requesting data from original literature sources after an extensive search for long‐term time series and a number of exploratory searches for time series to cover perceived gaps by theme, for example natural system and geographical region. In addition, data sets were processed to cover large geographical regions, for example continents and ocean basins, independently of global and hemispheric data that are often readily available. All acquired time series were processed in the same way. The resulting compilation of data was dependent on sampling, monitoring location and availability, and does not claim to be comprehensive or geographically representative of the whole world.

For inclusion in the analysed database, continuous data sets needed to be within the period 1946 to 2012 and start by 1980 at the latest. The year 1946 was chosen, when possible, as the start date to exclude the poor sampling during World War II. There were two exceptions, one to allow satellite information to be included using the Normalized Difference Vegetation Index (NDVI), start 1982 (Myneni *et al*., 1997) and the other mesopelagic fish eggs, start 1981 (Fujino *et al*., 2013).

#### Data sources

The 72 time series selected for more detailed study were chosen to yield as wide a global geographical coverage as possible; they include data from single sites, sets of data, and data averaged at global, hemispheric, continental and ocean scales. The data used in the study can be obtained from Table S1 or from the corresponding author. Citations for all the time series analysed are given in Table S2. The years from 1983 to 1990, between the major volcanic eruptions of El Chichón (1982) and Pinatubo (1991), were focused on as a ‘period of interest’ to study the 1980s regime shift.

Two data sources for temperature were used to derive continental, oceanic, global and hemispheric means. First, precalculated 5° grid annual mean anomalies with respect to 1961–1990 produced by the Hadley Centre of the UK Met Office and the Climatic Research Unit, University of East Anglia (http://www.metoffice.gov.uk/hadobs/index.html), were downloaded: HadCRUT4.3.0.0 (combined LST and SST); HadSST3.1.1.0 (SST); and CRUTEM4.3.0.0 (LST), the latter calculated from ~5500 monthly meteorological station temperatures. Second, monthly mean data were downloaded from the NASA Global Historical Climatology Network (GHCN) data set (ftp://ftp.ncdc.noaa.gov/pub/data/ghcn/v3). While the first set was prepared by the provider, with this data set we were able to preprocess the data ourselves to take into account gaps in sampling in time and space before calculating global, hemispheric and continental means.

#### Processing the GHCN global temperature data set

We used data from the GHCN data set to provide an independent temperature time series that is based on real and not gridded data. After preliminary investigations, the data set used for further study was limited to land meteorological stations from continental regions (6449 stations). The distribution of these stations was not random and did not systematically cover all regions of the world with a similar density, and many had missing data. To address these issues, the sequence of subsequent processing followed the order:

1. To reduce the number of stations with missing data, new regional mean time series were calculated for areas where adjacent weather stations were likely to show similar temperatures. A sensitivity analysis was carried out to check the similarity in data from stations enclosed within circles of diameter 50, 100, 150 and 200 km. A circle with a diameter of 100 km gave the best result, so ‘circle time series’ were calculated by averaging the data from stations within circles of this diameter.
2. To decide which stations to include within each circle time series, a threshold test was applied to filter the station time series for missing years. Seven options were tested: with 5, 10, 15, 20, 25 or 30 missing years, or with no filtering. A threshold with a maximum of 10 years’ missing data was selected, as using this threshold the signal was not altered by missing values.
3. For the calculation of annual means, at least nine sampled months were required, otherwise a missing value was attributed.
4. A final set of time series was then generated for each selected 100‐km‐diameter circle to give 302 circles in Europe, 624 in Asia, 228 in Australia, 97 in Africa, 1932 in North America and 108 in South America, with 2977 for the Northern Hemisphere, 314 for the Southern Hemisphere and 3291 for the world. Of these 3291 time series, the maximum number of circles that overlap is 37.
5. All the GHCN time series were anomalized with respect to their own mean over the period 1961–1990 for comparison with the Hadley and CRU time series.
6. A control sensitivity analysis was applied to determine whether any change in the number of circle time series per year coincided with observed regime shifts in the data. There were no major changes in the number of missing stations until 1989/1990 in some regions of the world and more globally in 2000; the former reduction in stations coincided with the break‐up of the USSR. This result gives increased confidence to the regime shifts identified in the GHCN data prior to 1989.

### Statistical analysis

#### Identifying regime shifts

Three different statistical methods were used to identify regime shifts.

**Figure 1:**
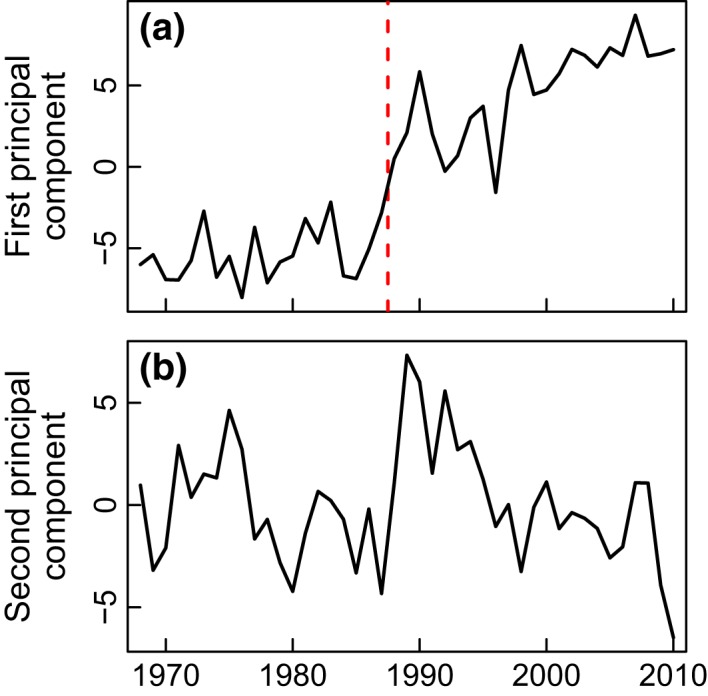
A standardized principal component analysis (PCA) of long‐term changes in 72 time series. (a) First principal component (49% total variance) and (b) second principal component (12% total variance). The red dashed line marks a significant regime shift year in 1987 (*P* ≤ 0.05), identified by both principal component and change‐point analyses.

First, a standardized principal component analysis (PCA) (Beaugrand *et al*., 2002) was applied to the whole data set. The period 1968 to 2010 was selected for the analysis as at least 58 of the 72 time series (80%) had data throughout this interval. PCA is not sensitive to temporal autocorrelation. Results of the analysis are shown in Fig. 1 and Table 1.

**Table 1:**
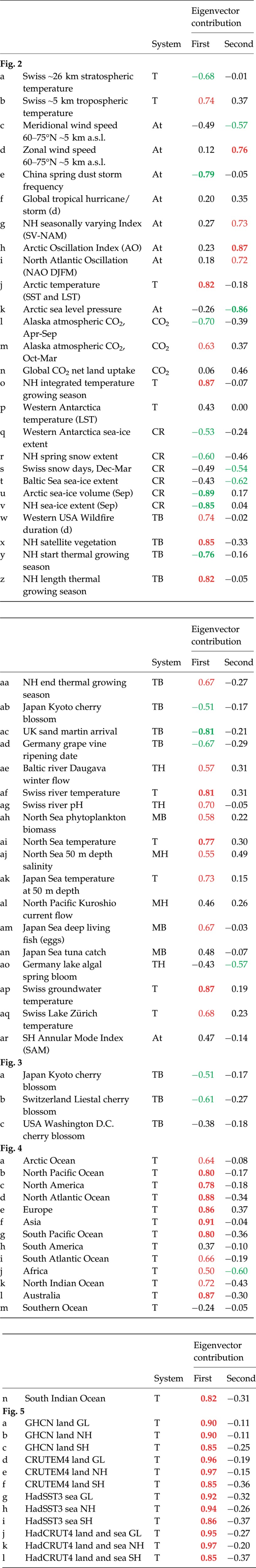
Standardized principal component analysis. Results of an analysis performed on the whole 72 time series as an entity for the period 1968–2010 where 80% of the time series had no gaps. The first two normalized eigenvectors show the correlation between each variable and the first two principal components. Colour code for eigenvector contribution: positive red 0.50*–*0.75, red bold 0.76*–*1; negative the same in green (See also Fig. 1). See Table 2 legend for 'system' acronyms

Second, we used a change‐point analysis (Taylor, 2000) to identify the timing of the shift in the first principal component; a technique that is simple, is not sensitive to outliers and takes into account the effects of temporal autocorrelation when calculating the probability of the shift. The cumulative sums of the time series (i.e. the first principal component) were calculated (Ibañez *et al*., 1993; Kirby *et al*., 2009); then, the first difference in the cumulative sums was estimated, followed by a Monte Carlo test to determine the probability that a shift had occurred based on the number of times the simulated first difference exceeded the observed amplitude. About 100,000 runs were performed, and the simulated time series were retained if their order‐1 autocorrelation was higher or equal to the one observed in the original time series.

**Figure 2:**
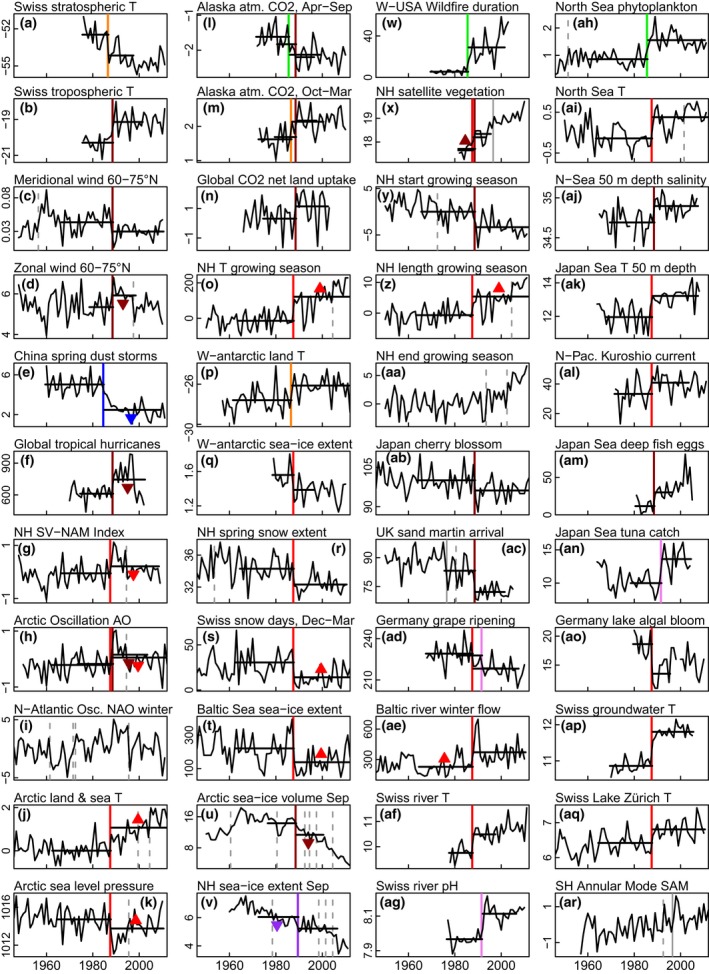
Significant regime shifts in time series representing a range of different Earth systems. Vertical lines denote regime shift years (*P* ≤ 0.05), coloured throughout the paper to reflect the ‘1980s period of interest’ (1983*–*1990): 1984 (blue), 1985 (green), 1986 (orange), 1987 (red), 1988 (brown), 1989 (purple), 1991 (pink as a lagged effect); grey solid lines mark the earlier and later regime shifts (1976 and 1996), and grey dashed lines other significant shift years outside the 1980s. Horizontal lines mark the longest test‐period with a significant result. Triangles (coloured as per the shift year) point up or down to indicate the direction of a significant trend before or after the shift year: here 3 before, 12 after the regime shift and 26 time series with a shift in the ‘period of interest’ but no trends. The plots are presented in the following sequence: atmosphere (a–p) and (ar), cryosphere (q–v), terrestrial biosphere (w–ad), hydrosphere (ae–aq). Further details for each of the time series are given in Tables 3 and S3 (origin, units and shift years).

**Figure 3:**
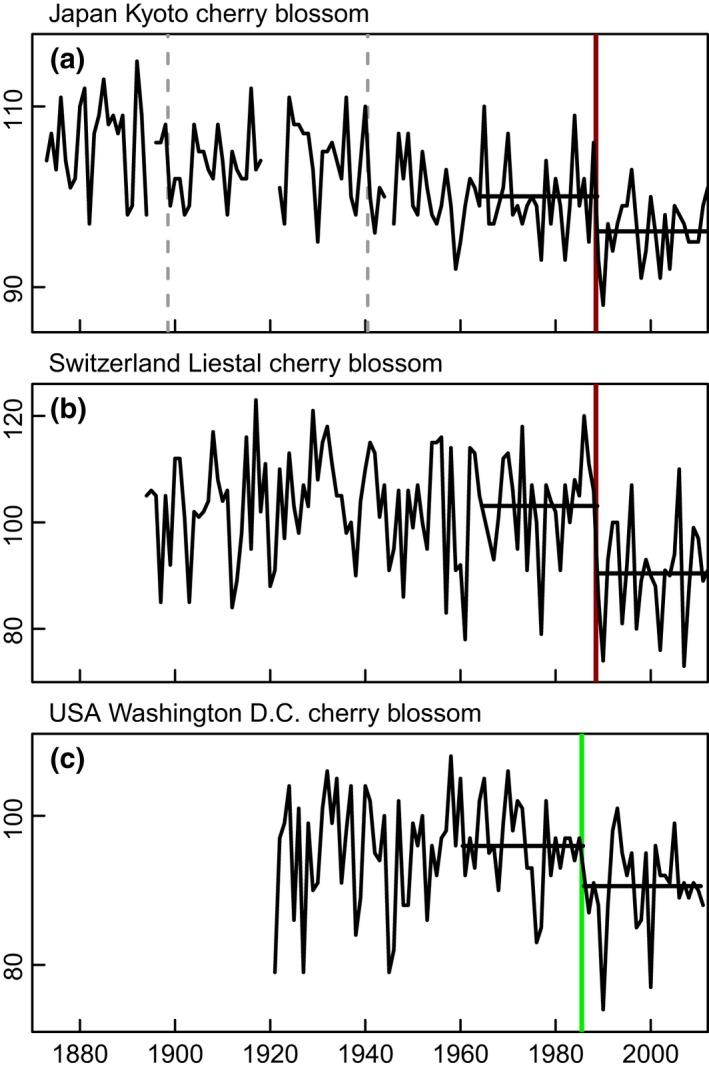
Long‐term time series of cherry blossom blooming dates from three different continents. Units: day of the year. Vertical lines denote significant regime shift years (*P* ≤ 0.05), coloured for the ‘1980s period of interest’ (1983*–*1990) throughout the paper, in this case: 1988 (brown) and 1985 (green); grey dashed lines mark other significant shift years outside the 1980s. Horizontal lines mark the longest test‐period possible with a significant result. (a) The date on which the blossom of the Japanese cherry (*Prunus jamasakura*) comes into full bloom in Kyoto, Japan, 35°N, 136.67°E (1873*–*2012). Data: updated and revised from Yasuyuki Aono. Shift years: 1898, 1940, 1988. (b) The start date of the flowering of blossoms on a cherry tree (*Prunus avium*) at Liestal, Switzerland, 47.48°N, 7.44°E (1894*–*2012). The tree is checked with a telescope every day at the beginning of the flowering season; the date when 25% of the blossoms are open marks the start. Data: Andreas Buser, Landwirtschaftliches Zentrum Ebenrain, Sissach and MeteoSwiss, Switzerland. Shift year: 1988. (c) The peak bloom date of the blossoms on the Yoshino cherry trees (*Prunus* x *yedonensis*) in the tidal basin, Washington D.C., USA, 38.88°N, 77.04°W (1921*–*2011). Peak bloom date is defined as the day when 70% of the blossoms on the trees in the basin are open. Data: from http://www.nps.gov/cherry/upload/Cherry-Festival-dates.pdf. Shift year: 1985.

**Figure 4:**
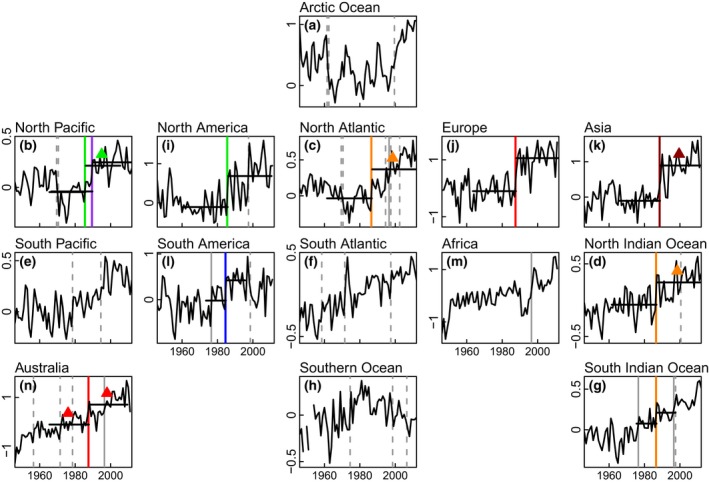
Time series of annual mean land/sea surface temperature for six continents and eight ocean basins. Anomalies calculated with respect to their own mean over the period 1961*–*1990 (continents 1946*–*2011, oceans 1946*–*2012). Vertical and horizontal lines, symbols and colours as per the legend for Fig. 2. Data for land from ftp://ftp.ncdc.noaa.gov/pub/data/ghcn/v3/, processed by Eric Goberville. Units: °C. (c) North America, shift years: 1985, 1997; (e) Europe: 1987; (f) Asia: 1988; (h) South America: 1976, 1984, 1998; (j) Africa: 1996; (l) Australia: 1956, 1971, 1978, 1987, 1996. Data for the sea processed by Jonathan Barichivich from http://www.metoffice.gov.uk/hadobs/hadsst3/data/download.html. All grid boxes with data for a given region were selected, averaged, and weighted by the cosine of the latitude to account for the changing size of grid cells towards the poles. Units: °C. (a) Arctic Ocean, shift years: 1961, 1962, 1999; (b) North Pacific Ocean: 1969, 1970, 1985, 1989; (d) North Atlantic Ocean: 1969, 1970, 1986, 1994, 1996, 1997, 2002; (g) South Pacific Ocean: 1978, 1994; (i) South Atlantic Ocean: 1958, 1971, 1997; (k) North Indian Ocean: 1986, 2000; (m) Southern Ocean: 1974, 1998, 2006; (n) South Indian Ocean: 1976, 1986, 1996, 1997.

**Figure 5:**
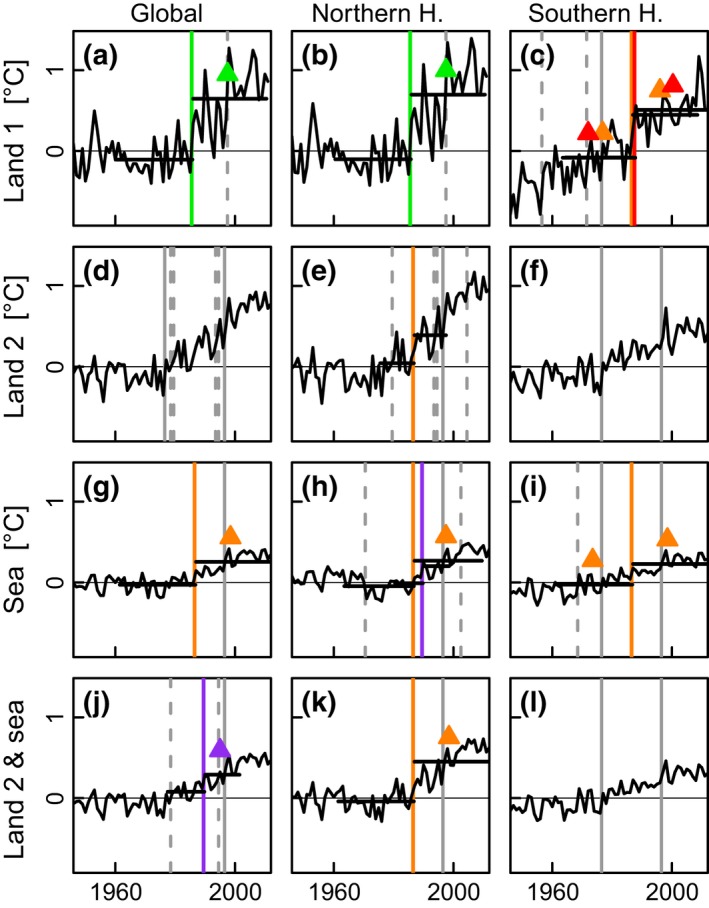
Time series of annual mean global and hemispheric land and sea surface temperatures. Periods: 1946*–*2011/2012. (a–c) GHCN land surface temperature. (d–f) CRUTEM4.3.0.0 land surface temperature. (g–i) HadSST3.1.1.0 sea surface temperature. (j–l) HadCRUT4.3.0.0 combined land and sea surface temperature. Vertical and horizontal lines, symbols and colours as per the legend for Fig. 2. All data series are anomalized with respect to their own mean over the period 1961*–*1990. Data: (a–c) Station‐based from ftp://ftp.ncdc.noaa.gov/pub/data/ghcn/v3. (d–l) 5° grid based from Hadley Centre, Met Office, UK http://www.metoffice.gov.uk/hadobs/ or from Climatic Research Unit, University of East Anglia, UK http://www.cru.uea.ac.uk/cru/data/. Units: °C. Shift years: (a) 1985, 1997; (b) 1985, 1997; (c) 1956, 1971, 1976, 1986, 1987; (d) 1976, 1978, 1979, 1993, 1994, 1996; (e) 1979, 1986, 1993, 1994, 1996, 2004; (f) 1976, 1996; (g) 1986, 1996; (h) 1970, 1986, 1989, 1996, 2002; (i) 1968, 1976, 1986, 1996; (j) 1978, 1989, 1994, 1996; (k) 1986, 1996; (l) 1976, 1996.

**Figure 6:**
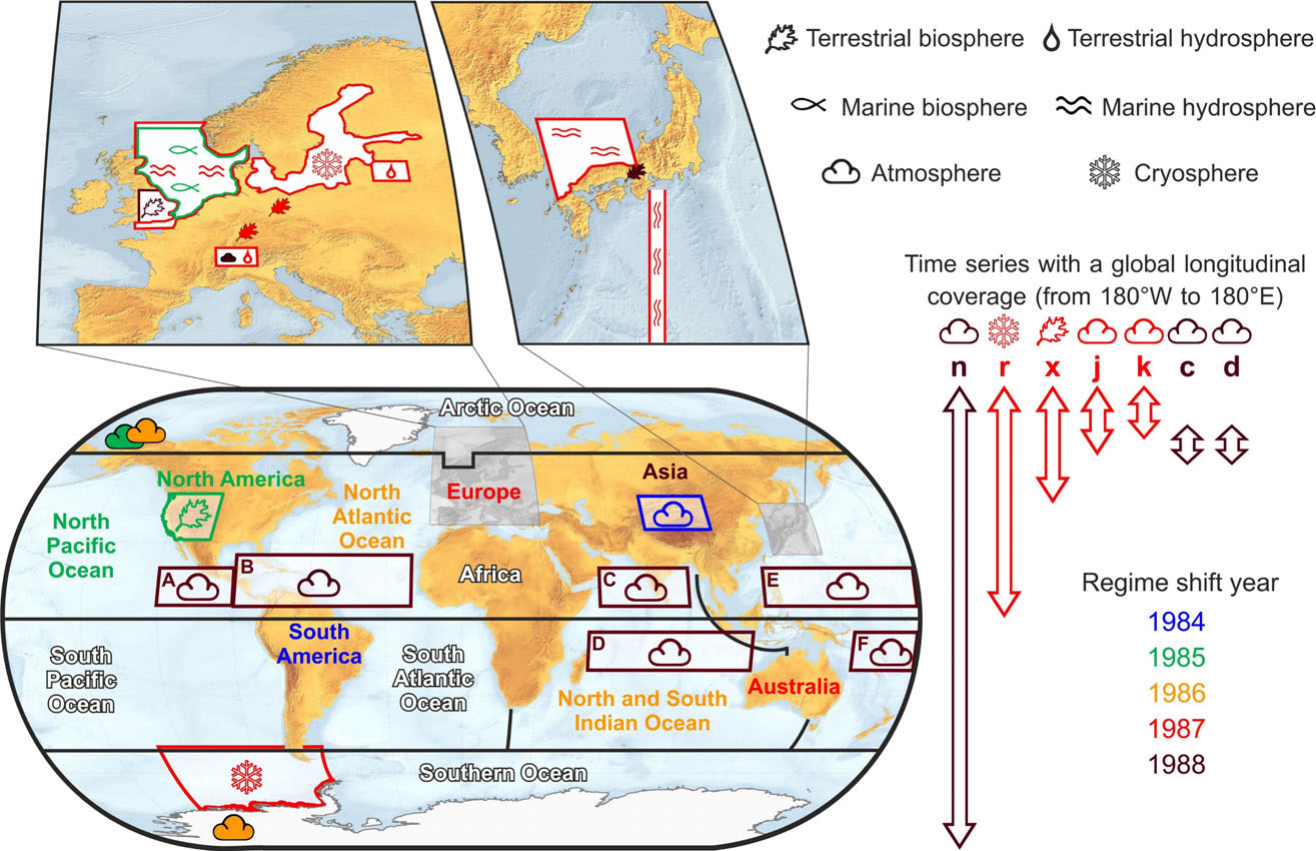
Global map with magnified insets: locations of time series plotted in Figs 2 and 4. The latitudinal extent of data averaged on a global or zonal basis is shown as arrows on the right (the letters above the arrows refer to the time series in Fig. 2). The time series are grouped into six system categories (symbols, top right); the terrestrial biosphere group also includes one freshwater biological category (Fig. 2ao). Individual sites are denoted by solid coloured symbols and regions by hollowed coloured symbols. The colours represent the regime shift year in each time series (see key, bottom right). White lettering with a black border (e.g. Africa) indicates time series with no significant regime shifts in the 1980s. The areas covered by the six regions averaged to produce ‘Global’ tropical storm days are labelled A to F (coordinates in Table S4). A list of the system allocation and shift years of each of the time series from Fig. 2 is given in Table S5; for oceans and continents see Fig. 4.

Third, for standardization and comparison of individual time series, a ‘multiple’ sequential *t*‐test analysis of regime shifts (STARS) based on the Rodionov method (Rodionov, 2004; Rodionov & Overland, 2005) was applied. This method was used individually on each time series; the results in the period of interest are given in Figs 2, 3, 4, 5, 6 and Table 2.

**Table 2:**
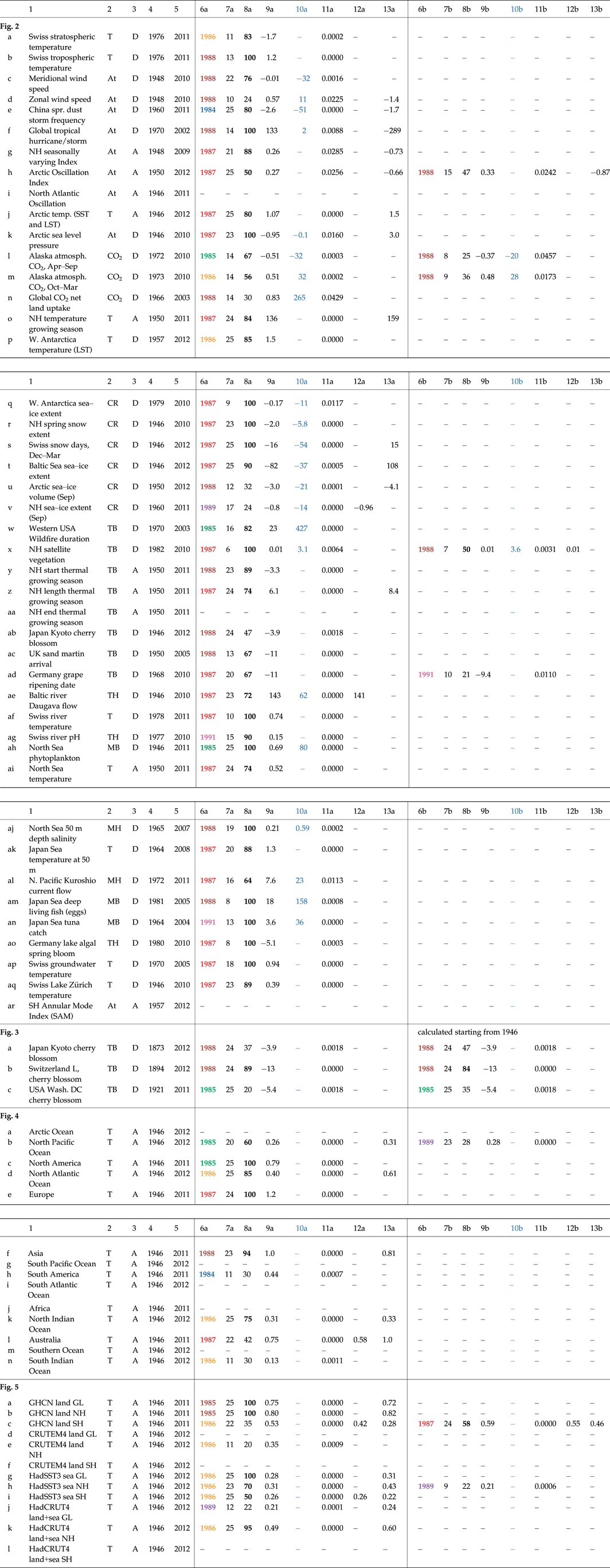
Results of the multiple sequential *t*‐test analyses of regime shifts (STARS) in Figs 2, 3, 4, 5 for the ‘period of interest’ (1983–1990). Introductory information is listed in columns 1–5. This is followed by two sets of results in sequence (columns 6a–13a and 6b–13b) as some of the 72 time series have two shift years in the ‘period of interest’. Introductory text: (1) brief title; (2) temperature/system category (T temperature, At atmosphere, TB terrestrial biosphere, MB marine biosphere, CO_2_ carbon cycle, CR cryosphere, TH terrestrial hydrosphere, MH marine hydrosphere); (3) whether the data are anomalies A or absolute values D; (4) the start year; and (5) the end year of the time series; (6a,b) the ‘shift year’ in bold to highlight the colours that are the same as in the legend for Fig. 2; (7a,b) the ‘longest test‐period’ [years] which gives a significant result; (8a,b) the strength of the change as ‘% significant results’, bold because it is a measure of the strength of the shift (A percentage is used here as it is not possible to include all the 20 default test‐periods in the calculation for short time series.); (9a,b) the ‘difference’ (in the units of each time series) as the size of the change between the means of the ‘longest test‐period’ to either side of the shift; (10a,b) the difference between the two means as a ‘% change’ where possible (not for temperature, anomalies, indices, day of year, pH); (11a,b) the ‘*P*‐value’ of the shift; (12a,b) the ‘trend old regime’ over the ‘longest test‐period’ prior to the shift; and (13a,b) the ‘trend new regime’ over the ‘longest test‐period’ after the shift

#### Variant of the Rodionov method

Generally, a *t*‐test examines whether the means of two sample populations differ significantly, based on the standard deviations and on the number of values in each sample. The existence of a trend in one or both samples is not excluded.

The STARS method (Rodionov, 2004; Rodionov & Overland, 2005) tests whether the end of one period (regime) of a certain length is different from a subsequent period (new regime). The cumulative sum of normalized deviations from the hypothetical mean level of the new regime is calculated, and then compared with the mean level of the preceding regime. A shift year is detected if the difference in the mean levels is statistically significant according to a Student's *t*‐test. In his third paper, Rodionov (2006) shows how autocorrelation can be accounted for. From each year of the time series (except edge years), the rules are applied backwards and forwards to test that year as a potential shift year. The method is, therefore, a running procedure applied on sequences of years within the time series.

The multiple STARS method used here repeats the procedure for 20 test‐period lengths ranging from 6 to 25 years that are, for simplicity (after testing many variations), of the same length on either side of the regime shift. The last year of the old regime is defined as the shift year, and all results shown are for *P* ≤ 0.05. Our approach allows us to assess the strength of a regime shift by the number of test‐period lengths with significant results, while Rodionov uses a regime shift index to weight his results.

Not all 20 test‐period lengths can be applied to all the time series as the length of the time series, and the location of the shift year in the time series can sometimes make this impossible. For example, it would not be possible to apply the longest test‐period length of 25 years to a short time series. For comparative purposes, we therefore determine the number of significant results as a percentage of the maximum possible, and set a minimum threshold of 20% to accept a significant shift year. This approach favours the detection of changes between long regimes and discards changes between short regimes, such as, for example, that in the NAO from 1989 to 1995, which lasted only 7 years (Fig. 2i).

Detection of a shift year depends very much on the test‐period used. Extreme values in a time series can create a barrier for short test‐periods and limit a shift year result to a small part of the whole time series. On the other hand, long test‐periods may integrate these values to give significant shift years for longer periods. These observations emphasize the importance of varying the test‐period length.

All acquired time series were analysed using the multiple STARS method. In some cases, because we used various test‐period lengths, two adjacent regime shift years were identified. Possible reasons for this duplication, other than the distribution of the values in the time series, include the following: that the shift event takes time to evolve over more than 1 year; that some examples of shifts occur in the last months of 1 year and in the first months of the next; and that several different shift years occur in a large area that has been averaged over.

#### A comparison of real data with artificial time series

To demonstrate that the identification of regime shifts in our analyses, based on the multiple STARS method, is not spurious, we compared the results obtained from the real time series with the results derived from two sets of artificial time series. The procedure used to create these autocorrelated time series is fully described in Beaugrand *et al*. (2014). First, we examined the 72 real time series with an autocorrelation function. The resulting autocorrelograms include information on the order if the correlation and the value for each lag, and showed a pronounced autocorrelation for some of our real time series (Fig. S1a). Second, the artificial time series were produced with two types of temporal autocorrelation, ‘high’ and ‘medium’, to approximate reality. For each type, 70 time series with 67 hypothetical years were simulated to correspond to the maximum period of the real time series (1946–2012, a total of 4690 years each). In the real data, some of the 72 time series extended over a shorter period (a total of 4104 years or 88% of the maximum possible).

The two types of autocorrelation were as follows: (1) High temporal autocorrelation, where 70 time series were constructed with a linear trend of magnitude 100 (arbitrary units) and random temporal fluctuations of magnitude 120 (green in Fig. S1b). These time series had an autocorrelation that corresponded to the maximum autocorrelation observed in the real time series (upper fine lines in Fig. S1a). (2) Medium temporal autocorrelation, where 70 time series were constructed with a linear trend of magnitude 100 (arbitrary units) and random temporal fluctuations of magnitude 45 (blue in Fig. S1b). These time series had an autocorrelation that corresponded to the medium autocorrelation in the observed time series (central fine lines in Fig. S1a). The thresholds of 45 and 120 were chosen to correspond to the two main situations (i.e. high and medium levels of autocorrelation) encountered in the time series. The total number of significant test‐periods and shift years for the real and the two artificial time series sets are shown in Fig. S2.

A further comparison between the results for the real and artificial data sets shows the longest significant test‐periods observed (Fig. S3a–c), where the triangular shape of the plots reflects the longest possible test‐period length at the edges of the time series, from 6 years at the bottom to 25 years at the top. The strength of shift years in a specific calendar year is shown as a percentage of the number of significant test‐periods against the total number of possible test‐periods (Fig. S3d–f). In the middle of long time series the latter is 20 test‐periods, but at the edges it is less. A cut‐off of 20% was applied to eliminate the majority of only one or two significant test‐periods, which are more likely to be artefacts.

### Modelling

#### CMIP5 historical climate modelling

Global mean historical CMIP5 temperature data (Jones *et al*., 2013) were used to attribute the changes in observed global temperature from the HadCRUT4.3.0.0 data set (Morice *et al*., 2012). Two multimodel ensembles were selected, firstly with natural (solar and volcanic) forcings (with a total of 46 members), and secondly with both natural and anthropogenic forcings (99 members). The multimodel ensemble mean is taken to represent the best estimate of the response of global mean temperature to the forcings in each ensemble. The difference between the two ensemble means is used as an estimate of the effect of anthropogenic forcings on global mean temperature. Linear least‐squares fitting was used to obtain running 7‐year trends in the various global mean time series.

#### Statistical modelling

Estimates of the response of global mean temperature to solar and volcanic forcings individually were produced based on statistical reconstructions (Folland *et al*., 2013). This latter study used a cross‐validated multiple regression approach on data from 1891 to 2011 to estimate the effect of a range of known influences on global mean temperature. The sum of the solar and volcanic reconstructions was calculated to provide an equivalent to the ensemble mean temperature in the CMIP5 natural forcing ensemble. Calculation of anomalies and trends was performed using similar techniques to those used for the CMIP5 data.

## Results

### Identification of the 1980s regime shift in a wide range of Earth systems

Many statistical techniques exist to identify regime shifts (e.g. Beaugrand, 2004; Mantua, 2004; Rodionov & Overland, 2005; Rodionov, 2006; Beaulieu *et al*., 2012a; Varotsos *et al*., 2013). All have strengths and drawbacks, but the three we use here are complementary. To determine the main long‐term patterns of variability, we applied a standardized principal component analysis (PCA) (Beaugrand *et al*., 2002) to the data set as an entity. The results (Fig. 1, Table 1) showed a clear shift in 1987 for the first component, which accounts for almost half (49%) of the total variance. A large number of time series, especially of variables related to temperature and vegetation, are strongly correlated with the first component. The second component, which accounts for 12% of the total variance, is correlated, for example, with the Arctic Oscillation, North Atlantic Oscillation, Arctic sea‐level pressure and zonal wind. We applied Taylor's change‐point analysis (Taylor, 2000) to identify stepwise shifts along the first principal component (Fig. 1a); the analysis detected a significant regime shift in 1987 (*P* ≤ 0.05).

For standardization and a more detailed analysis of regime shifts in individual time series, the multiple version of the STARS method (Rodionov, 2004, 2006; Rodionov & Overland, 2005) described above (see Materials and methods) was employed. The well recognized and documented Rodionov method has now been used in several dozen papers (e.g. Luczak *et al*., 2011; Jaffré *et al*., 2013; Litzow & Mueter, 2014). Multiple STARS results for 44 time series representative of six natural systems (terrestrial and marine biospheres/hydrospheres, the atmosphere and cryosphere) are shown in Fig. 2, with more information on the time series given in Table 3 and Table S3. The multiple STARS method identifies a shift from a mostly stationary state to a new state, which in a third of cases contains a trend after the regime shift (Table 2). Note that our definition allows a significant linear trend within a regime.

**Table 3:** Summary details of the time series presented in Fig. 2. Additional information in Table S3

a‐b	°C	Stratospheric air temperature measured by radiosondes launched at Payerne, Switzerland. (*a*) at 20 hPa (~26 km above sea level), (*b*) at 500 hPa (~5 km above sea level)
c	m s^−1^	Meridional wind speed at 500 hPa
d	m s^−1^	Zonal wind speed at 500 hPa
e	No. of days yr^−1^	Number of spring (March ‐ May) dust storms per year, averaged for 48 observing stations in northwest China
f	No. of days yr^−1^	Number of tropical cyclone storm days per year (hurricanes plus tropical storms)
g	‐	Seasonally varying Northern Hemisphere annular mode (SV‐NAM) index
h	‐	Arctic Oscillation (AO) index: the dominant pattern of winter (Nov‐Apr) sea level pressure variation north of 20°N
i	‐	North Atlantic Oscillation (NAO, December ‐ March) index: a basin‐scale alternation of atmospheric mass over the North Atlantic between high pressure in the subtropical Atlantic and low pressure around Iceland
j	°C	Combined land‐surface air temperature and sea‐surface water temperature for the zonal band >64°N. Anomalies relative to 1951–1980
k	hPa	Sea‐level pressure from the 20th Century Reanalysis version 2 data averaged over the Arctic
l‐m	ppm	Interannually detrended atmospheric CO_2_ concentration at Point Barrow, Alaska: (*l*) during the warm (April ‐ September), and (*m*) during the cold (October ‐ March) seasons
n	Pg C yr^−1^	Global net CO_2_ land uptake
o	°C	Time‐integrated air temperature over the thermal growing season for the extratropical (>35°N) Northern Hemisphere. Thermal growing season if temperature >5 °C
p	°C	A reconstructed time series of air surface temperature for the Byrd meteorological station in Western Antarctica
q	10^6^ km^2^	Sea‐ice extent in the Bellingshausen/Amundsen Seas, Western Antarctica
r	10^6^ km^2^	Northern Hemisphere spring (March–April) snow extent
s	Snow days	Number of snow days on the north side of the Swiss Alps between December and March
t	10^6^ km^2^	Maximum sea‐ice extent in the Baltic Sea and Kattegat
u	10^3^ km^3^	Modelled total sea‐ice volume in September for the Arctic Ocean
v	10^3^ km^2^	Northern Hemisphere (September) sea‐ice extent
w	No. of days	Mean number of burning days per wildfire event in forests of the western USA
x	–	Normalized Difference Vegetation Index (NDVI) averaged for the growing season in northern latitudes (>35°N)
y‐aa	Day of the year/days	Thermal growing season (temperature >5 °C) for the extratropical (>35°N) Northern Hemisphere, (*y*) start (day of the year), (*z*) length (days), (*aa*) end (day of the year)
ab	Day of the year	The flowering date on which the Japanese cherry, *Prunus jamasakura*, comes into full bloom in Kyoto, Japan. A plot of the time series starting in 1873 is shown in Fig. 3
ac	Day of the year	Mean first arrival dates of the sand martin, *Riparia riparia*, averaged for eight locations in the UK
ad	Day of the year	Véraison (colour change and initial maturation) of Müller‐Thurgau grapes harvested from vineyards in Franconia, Germany
ae	m^3^ s^−1^	Winter flow (December–February) in the >1000 km long River Daugava measured at the Daugavpils hydrological station in Latvia
af	°C	River water temperature averaged for 18 hydrological stations that are representative of >80% of the river outflow from Switzerland
ag	–	River water pH averaged for 6 hydrological stations that are representative of >80% of the river outflow from Switzerland
ah	Colour categories	Phytoplankton biomass: a visual estimate of chlorophyll sampled at ~10 m depth by the Continuous Plankton Recorder and averaged for the North Sea
ai	°C	Temperature of the North Sea averaged for the full depth within grid cells with centres enclosed by 50°N‐61°N and 3°W‐9°E, anomalies relative to 1971–2000
aj	–	Mean salinity at 50 m depth at the deepest station on the Torungen–Hirtshals hydrographic section between Norway and Denmark in the North Sea, Skagerrak
ak	°C	Sea temperature at 50 m depth based on monthly measurements taken in the Japan Sea
al	10^6^ m^3^ s^−1^	Mean summer (July–September) volume transport of the Kuroshio Current in the Western North Pacific
am	No. of eggs m^−2^	Egg abundance per m^2^ of the deep water (mesopelagic) fish *Maurolicus japonicus* sampled by regular ichthyoplankton net surveys in May in the eastern Japan Sea. The eggs are found below 100 m and are used as an index of the biomass of the adult population
an	10^3^ tons	Tuna catch in Japanese waters of the Japan Sea
ao	Calendar week	Timing of the spring algal bloom in Lake Müggelsee, Berlin, Germany
ap	°C	Mean groundwater temperature of two Swiss aquifers fed by riverbank filtration
aq	°C	Volume‐weighted mean temperature of Lower Lake Zurich, Switzerland
ar	–	The Southern Annular Mode (SAM) index is a zonal feature that reflects the main variability of atmospheric circulation in the Southern Hemisphere extratropics and high latitudes

The results of the multiple STARS method stress again the importance of the 1980s event compared to the smaller regime shifts in the 1970s and 1990s. The dominant 1980s regime shift also shows synchronous timing for systems and geographical regions. A total of 165 step changes were identified in the 72 time series over the analysis period from 1946 to 2012. Of these, 11% occurred in the late 1970s (1973–1980), 40% in the late 1980s (1983–1990) and 25% in the late 1990s (1993–2000). A comparison between the scale of the 1970s, 1980s and 1990s regime shifts can also be made by noting the maximum hemispheric step change in temperature shown by the GHCN LST time series with differences between the old and new regimes of 0.38 °C in 1976 (Southern Hemisphere), 0.80 °C in 1985 (Northern Hemisphere, Table 2) and 0.66 °C in 1997 (Northern Hemisphere).

Within the ‘1980s period of interest’ (1983–1990), 66 step changes were found in 59 of the 72 time series (82%). In some of these, none were found, and in others more than one. Of the 66 detected shift years, very strong shifts (100% of the possible test‐periods significant) were found in 27% and strong shifts (≥50% of the possible options) in 74%, the latter divided into 35% and 39% between temperature (for all air, sea and freshwater data sets) and a grouping of all other time series (36 each). Of the 44 time series presented in Fig. 2, 41 show a step‐change in the mid‐1980s, only two (Fig. 2v, ac) in the mid‐1970s, and only 11 (Fig. 2d, j, k, u, v, x, aa and all the indices Fig. 2g, h, i, ar) in the mid‐1990s. The timing of the 1980s event in all natural systems ranges from 1984 to 1989, with most changes occurring in 1987 or 1988. Two examples are shown where the timing of the shift may reflect a lagged response (Swiss river pH and tuna catch in the Japan Sea, Fig. 2ag, an).

The change can be expressed as a percentage for 24 of the 72 time series (not for temperature, anomalies, indices, day of year or pH). In 19 of these 24 time series, the change between the two levels of the longest test‐period with a result was ≥10%, in 7 ≥ 50% and in 3 ≥ 100%. In one – wildfire duration in the western USA – there was an increase of >400%. This means, from our calculations, that Western US wildfires lasted on average 29 days between 1986 and 2003 compared to 5.5 days between 1970 and 1985 (Table 2).

If the regimes (of *n* years length and for the longest test‐period with a result) to either side of the shifts are considered, with the term ‘old’ applied to the period before and ‘new’ to that after the regime shift, there are few significant trends in the old regimes, but many more in the new, emphasizing the dynamic nature of the change after the 1980s. Significant trends in the old regimes before the shifts were only found in 11% of the 66 shifts in the ‘period of interest’ and in the new regimes after the shifts 41%, while 55% had no significant trends either before or after the shifts. Only three time series (Australia, Fig. 4l; GHCN land SH, HadSST3 sea SH, Fig. 5c,i) show trends in both old and new regimes, which means for these three out of 72 another method would be more appropriate.

To demonstrate that the identification of regime shifts determined by the multiple STARS method is not spurious and to address autocorrelation, we compared the results from the real data with those from two sets of artificial time series (Figs S1–S3). The sum of significant test‐periods or shift years in the ‘1980s period of interest’ is much greater in the real time series than achieved by the two sets of artificial time series throughout the analysed time period and conclusively confirms the reality of the 1980s regime shift and the validity of the multiple STARS method. In the real data, a total of 165 shift years were detected with 40% in the ‘period of interest’; in the data sets with high and medium autocorrelation, the number of shift years were 348 and 172, of which only 16% and 12%, respectively, occurred in the ‘period of interest’. We expected that the shift years would be evenly distributed over the analysed time period in the artificial time series and more concentrated in the real data, and this is clearly evident. The probability of detecting a spurious shift is thus likely to be the same in all of the time series. Both sets of the artificial time series have a symmetrical pattern to either side of the middle of the analysed time period (Fig. S3b, c, e, f), whereas the real data are strongly biased towards the second half of the time series and especially within the ‘period of interest’ (Fig. S3a, d). An edge effect is evident in all the plots, originating in the start and end phase of the time series where shift years cannot be detected.

A long‐term context for the 1980s shift is provided by three centennial‐scale time series of the flowering date of cherry trees in Japan, Switzerland and the USA (Fig. 3). The 1980s step changes at Liestal in 1988 and Washington in 1985 are the only significant shifts in these time series in at least 80 years. Flowering date calibrated against springtime air temperature showed that the earliest timing of the bloom and warmest period in over 1000 years in Kyoto (Aono & Kazui, 2008) followed the shift in 1988.

In continental averages of LST (Fig. 4c, e, f, h, j, l), the 1980s regime shift is evident for all continents except Africa, ranging from 1984 in South America to 1988 in Asia. However, for mean oceanic SST (Fig. 4a, b, d, g, i, k, m, n), the shift is only shown for the North Pacific in 1985, the North Atlantic in 1986 and the south and north basins of the Indian Ocean in 1986. For both LST and SST, the evidence for shifts in other decades is limited. Our analyses confirm earlier findings that SST has increased in steps over the last century (Reid & Beaugrand, 2012; Varotsos *et al*., 2013) and that the pronounced upward trend in global combined land and sea temperatures seen in the decadal means of Figure SPM.1 in the IPCC Summary for Policy Makers (IPCC SPM, 2013; see also Fig. S4) started in the 1980s. Land and sea surface temperatures averaged over the entire globe and over the Northern and Southern Hemispheres separately (Fig. 5) record rapid increases as significant regime shifts centred on approximately 1976, 1986 and 1996. The relative increase in temperature is much greater over land than over the sea, and also larger over the Northern Hemisphere than over the Southern Hemisphere. The 1970s regime shift was first described for the North Pacific (Hare & Mantua, 2000), but in our results is evident only in the Southern Hemisphere. By contrast, the 1980s and 1990s regime shifts are seen in both hemispheres, although the 1980s event is stronger in the Northern Hemisphere.

The atmosphere underwent a major transformation around 1988 (Lo & Hsu, 2010; Xiao *et al*., 2012), with changes in temperature, meridional and zonal wind patterns, and pressure (Fig. 2a–f, j, k, o, p). Changes in zonal winds, tropical storms and arctic sea level pressure during the 1980s regime shift (Fig. 2d, f, k) and a subsequent reversal, in part associated with the 1996 regime shift (Xiao *et al*., 2012), reflect a pattern that can also be seen in the dominant general circulation modes in the Northern Hemisphere (Fig. 2g–i). However, the 1980s regime shift appears unique in that it does not show a relationship with El Niño (Yasunaka & Hanawa, 2003).

Many step changes in the 1980s are apparent in cryospheric records (Fig. 2q–v). In West Antarctica and the Arctic, an approximately synchronous regime shift in sea‐ice extent occurred in the 1980s, with modelled sea‐ice volume in the Arctic declining linearly by 21% from 1989 to 2012 (Fig. 2u). In marine systems the 1980s regime shift is well documented (Möllmann & Diekmann, 2012) (Fig. 2ah‐an) and coincided with the Eastern Mediterranean Transient in 1987, which initiated profound hydrographic changes that appear to be unique in the last hundred years (Roether *et al*., 2014).

Measured since 1982, satellite observations of vegetation greenness/plant biomass (Normalized Difference Vegetation Index, NDVI) reveal a step increase in plant growth in the Northern Hemisphere in 1987/88 (Fig. 2x). This index, derived from Advanced Very High Resolution Radiometer (AVHRR) satellite imagery, is a measure of the photosynthetically active radiation absorbed by chlorophyll in the leaves of plants. Other satellite‐ and ground‐based observations of the start and length (Fig. 2y, z), but not the end (Fig. 2aa), of the vegetation growing season and the cumulative temperatures over this period (Fig. 2o) show that the increase in productivity has closely tracked the rate of warming in the northern extratropics (Barichivich *et al*., 2013) and associated reductions in snow cover (Fig. 2r, s). This evidence is reinforced by additional extensive *in situ* measurements of changes in vegetation composition and biomass (Sturm *et al*., 2001; Mann *et al*., 2012; Brandt *et al*., 2013) and by other synchronous changes in terrestrial and freshwater systems (Fig. 2ab‐ag, ao‐aq).

Annual emissions of CO_2_ from fossil fuel and land use sources either accumulate in the atmosphere (atmospheric growth rate) or are removed from the atmosphere and absorbed by the land and oceans (sinks). The net land uptake is calculated from emission data, minus the atmospheric CO_2_ growth rate and minus modelled ocean uptake. A rapid increase in photosynthetic activity in the late 1980s is consistent with the step decrease in summer atmospheric CO_2_ concentration in northern latitudes and the simultaneous increase in winter atmospheric CO_2_ (Fig. 2l, m) (Barichivich *et al*., 2013), the latter a likely consequence of increased ecosystem respiration (Barichivich *et al*., 2012). This linkage is confirmed by carbon cycle observations and models (Sarmiento *et al*., 2010) that show a sudden increase of ~1 Pg C yr^−1^ in net uptake by the land around 1988 (Fig. 2n). The regime shift coincided with a sudden decline in the annual growth rate of atmospheric CO_2_ (Beaulieu *et al*., 2012b). This large increase in the net land carbon sink is evident in the global carbon budget despite increased carbon emissions from anthropogenic sources and from greater fire activity (Fig. 2w).

The PCA and the change‐point analysis results suggest that the main shift took place in 1987. The 72 individual time series show shifts within the ‘period of interest’ in the proportions 1983 (0), 1984 (2), 1985 (8), 1986 (12), 1987 (23), 1988 (17), 1989 (4), 1990 (0) = 66, with three shifts in 1991 that are likely to be lagged effects. Regionally, the results are generally closely grouped and show a degree of consistency with regard to the year of their occurrence. For example, in North America: wildfires, atmospheric summer CO_2_ concentration, the Washington Cherry and the mean LST all showed a shift in 1985; and in Europe eight time series from the cryosphere, fresh water and vegetation systems all showed a shift in 1987, with a shift in a ninth series, the UK sand martin arrival in 1988. These regional differences are mapped in Fig. 6 by ocean and continent for SST and LST (Fig. 4) with superimposed shift years for some of the time series plotted in Fig. 2. With the exception of dust storms in Asia (Fig. 2e), the signal seems to have started in the early 1980s in South America (1984), spreading to the North Pacific and North America (1985), to the North Atlantic Ocean (1986) and Europe (1987), and then on to Asia (1988), with a possible, weaker, second signal in the North Pacific in 1989 (see Fig. 4b and Table 2). In the Southern Hemisphere, it seems to have extended eastwards to the Indian Ocean (1986) and to Australia (1987).

### Using CMIP5 and statistical modelling to examine mechanisms

**Figure 7:**
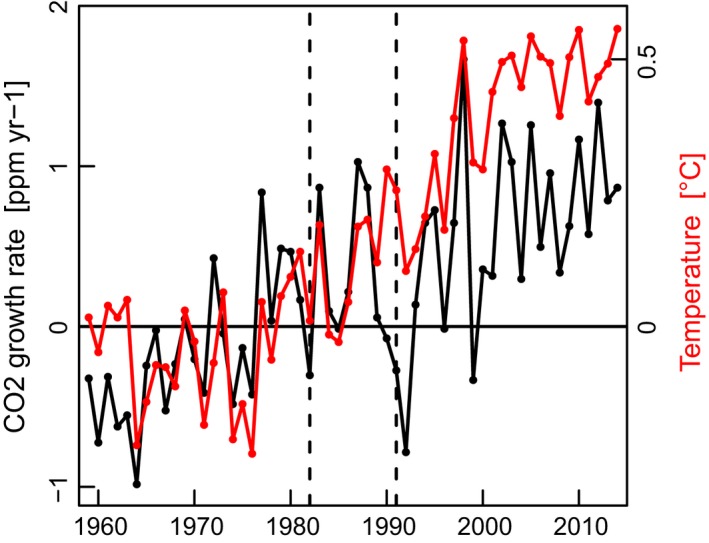
Annual temporal development of CO_2_ growth rate and global temperature. Correlation: (*r* ^2^ = 57%, *P* < 10^−10^). Period: 1959*–*2014. Mauna Loa CO_2_ annual growth rate (black) plotted against HadCRUT4.3.0.0 annual global combined land and sea surface temperature (red), both as anomalies to 1961*–*1990. Mauna Loa data from: ftp://aftp.cmdl.noaa.gov/products/trends/co2/co2_gr_mlo.txt and Hadley data from: http://www.metoffice.gov.uk/hadobs/hadcrut4/data/current/download.html.

The global scale of the 1980s shift documented here suggests that a fundamental shift in the climate system took place at this time. It is clear that rapidly increasing temperature is central to the shifts, with rising concentrations of greenhouse gases contributing to a net warming of global climate since the late 1970s (Figure SPM 1, in IPCC SPM, 2013). Temperature is also important for the carbon cycle as well as *vice versa,* as reflected in the correlated changes in global combined land and sea surface temperature and the atmospheric growth rate of CO_2_ over the last ~50 years (Fig. 7).

**Figure 8:**
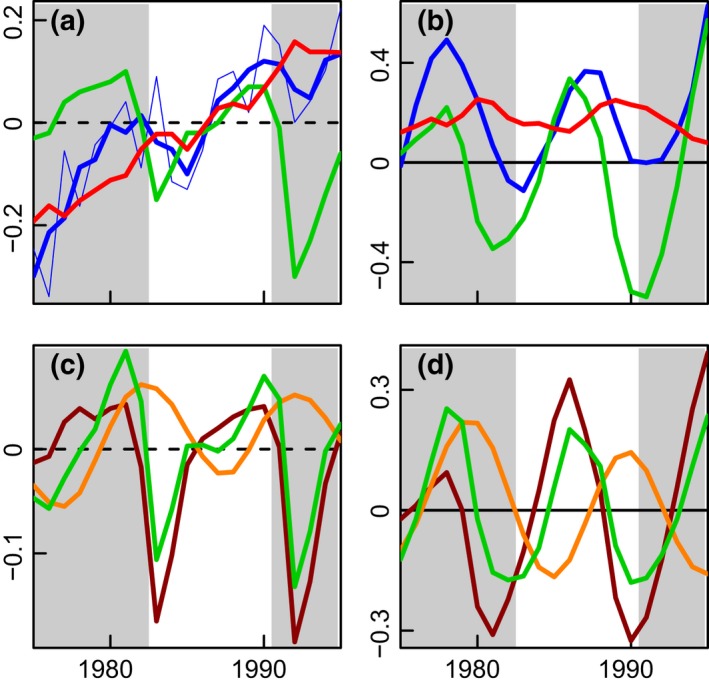
Attribution of the global mean temperature shift in the 1980s. The ‘period of interest’ (1983*–*1990) is highlighted in white. (a) Observed annual global mean temperature [°C] (blue fine) with its centred running 3‐year means (blue bold), and the response [°C] to anthropogenic (red) and natural (green) forcings from CMIP5 climate model simulations. (b) Centred running 7‐year trends [°C per decade] corresponding to the variables in (a). (c) Statistical reconstructions of the impact of solar (orange) and volcanic (brown) forcings on global mean temperature, plus their total (green). (d) Centred running 7‐year trends [°C per decade] corresponding to the variables in (c). All data series are anomalized with respect to their own mean over the period 1985*–*1988. In reading the figure, note that the RUNNING means as well as the RUNNING trends begin per definition to rise/decline before the El Chichón eruption starts.

Over the period 1975 to 1995, the anthropogenic warming from CMIP5 simulations of 0.19 °C per decade is close to the observed warming rate of 0.16 °C per decade (Fig. 8a). With a relatively steady trend (Fig. 8b), however, it does not explain the abrupt and substantial temperature shift in the 1980s. Natural forcing, in contrast, induced a marked peak in short‐term warming of 0.34 °C per decade in 1986 (in the 7‐year trend belonging to the period 1983 to 1989, Fig. 8b), temporarily exceeding the anthropogenic warming rate. Therefore, anthropogenic and natural forcing factors combined in the mid‐1980s to produce a sudden acceleration in global warming.

Statistical modelling of historical temperatures (Folland *et al*., 2013) indicates that this large natural forcing has a volcanic origin rather than being linked to solar radiation, the other possible natural forcing factor (Fig. 8c,d). In the ‘period of interest’, the statistically derived natural forcing has a similar pattern to that from the CMIP5 model simulations but is somewhat smaller in amplitude (Fig. 8b,d).

The major volcanic eruption of El Chichón in 1982 was responsible for an estimated cooling of 0.2–0.3 °C per decade, offsetting anthropogenic warming and resulting in relatively small global mean temperature trends in the early 1980s. By the mid‐1980s to late 1980s, however, recovery from the climatic impacts of the eruption, including a reduction in stratospheric aerosol concentrations, led to a natural warming, reinforcing anthropogenic warming and producing a rapid increase in global mean temperature on a higher level than before the eruption (Fig. 7). As a result, the global climate shifted to a warmer state in just a few years, setting in motion a cascade of responses in natural systems.

## Discussion

Based on evidence from a wide range of Earth system components, we show that a global and approximately synchronous shift occurred in the 1980s that is strongly evident in global, hemispheric and some local temperatures. Since the regime shift, decadal temperatures have shown a steep increase compared to the previous period of little change and an earlier smaller rise from 1920 to 1950 (Fig. S4).

Temperature appears to be the main forcing factor behind the shift: it is fundamental to most chemical, physical and biological processes. Independent of our study, there is substantial evidence that changes in the heat structure of the world are profoundly affecting regional climate variability, the cryosphere, terrestrial systems, sea level, ocean hydrodynamics and the biogeochemistry, ecosystems and living resources of the world. In a biological context, temperature modulates all processes, including the physiology, reproduction, development, occurrence, behaviour, disease and phenology of organisms, at cellular to ecosystem scales. The changes in temperature show great similarity across the world; this commonality provides a plausible explanation for the synchrony of the changes we observed. The abrupt increase in temperature during the regime shift may have initiated the intensification of environmental impacts, for example storms, floods, forest fires and the spread of pests seen over the last few decades.

The increase in temperature of the 1980s has also been linked to diverse biological changes on tropical mountains of the New World. In a Costa Rican cloud forest, declines of amphibian and reptile populations and shifts in the altitudinal distribution of birds are associated with a decrease in mist frequency and other local climatic changes that appear to have crossed an important biological threshold in 1987 (Pounds *et al*., 1999). Extinction of harlequin frog species across Central and South America, which, along with the disappearance of the golden toad from Costa Rica, have been associated with disease outbreaks and were the first species‐level extinctions in which global warming was implicated, accelerated in the mid‐1980s (Pounds *et al*., 2006).

The numerous individual processes behind sudden changes (regime shifts) in a temporal context as defined in this study are still poorly understood and to our knowledge have not been replicated in climate models (Lo & Hsu, 2010). However, analyses using CMIP5 model scenarios and patterns of global temperature and precipitation have been carried out and estimate that pronounced geographical changes will occur in climate regimes and vegetation types by 2100 (Feng *et al*., 2014). This study of vegetation types confirms the key role of temperature, highlights the regional importance of precipitation and emphasizes the large size and potential impact of future change (a ~31 to 46% increase in warmer and drier climate types by 2070–2100 based on RCP4.5 and RCP8.5 scenarios).

The long and precipitous decline in the CO_2_ growth rate in the period after 1988 (Keeling *et al*., 1995; Fig. 7), despite higher temperatures in 1989/90, coincides with the step increase in the terrestrial carbon sink (Fig. 2n). This decline in the CO_2_ growth rate and the net increase in the terrestrial sink from 1988 started well before the Pinatubo eruption in 1991. The sink was mostly in northern temperate boreal regions, as increases in the tropics were counterbalanced by deforestation and changes in land use (Sarmiento *et al*., 2010). Likely reasons for the enhanced biospheric carbon sequestration after the regime shift are increased photosynthesis from CO_2_ fertilization, an earlier and longer thermal growing season in the Northern Hemisphere (Fig. 2o, y, z), expansion of forests (IPCC Chap. 6, 2013) and the increase in surface solar radiation known as global brightening (Wild, 2009). To quantify approximately the change in the carbon cycle, decadal means from table 4 of Le Quéré *et al*. (2013) were used to calculate averages for the periods 1960 to 1989 and 1990 to 2009 of three variables: total anthropogenic emissions, atmospheric growth rate and carbon uptake by the combined land and ocean sinks (Table 4). The rate of carbon uptake by the combined land and ocean sinks (carbon storage) increased between these periods by ~1.65 Pg C yr^−1^, a 52% increase over the earlier period. To place this regime shift in carbon storage in context, it is close to double the rise in atmospheric growth rate of CO_2_ (0.92 Pg C yr^−1^) and equals 64% of the increase in total anthropogenic emissions between the same periods (2.57 Pg C yr^−1^) (Le Quéré *et al*., 2013). If a sink of this magnitude reversed to a CO_2_ source, it would markedly accelerate the rise in atmospheric CO_2_ growth rate and temperature.

**Table 4:**
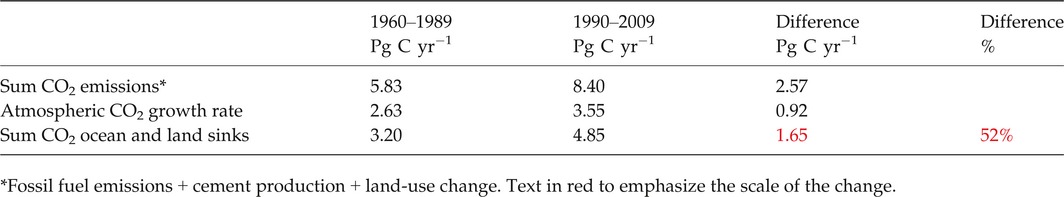
Change in CO_2_‐sink after the 1980s regime shift. Based on data from table 4 in (Le Quéré et al. (2013)

The mechanisms behind the apparent easterly movement seen in the timing of the 1980s regime and possible interhemispheric transfer of the year of the shift (e.g. South to North America) around the world are not understood. The pattern is best seen in the Northern Hemisphere where it in part alternates between land and ocean, starting in the Pacific in 1985 to Asia in 1988 (Fig. 4), reflecting the dominant flow of mid‐latitude winds and possibly the progressive movement of waves along the eastward flowing polar and subtropical jet streams.

What is the trigger that initiated the sudden increase in temperature from the 1980s until 2010 (Fig. S4)? Did the Earth's climate jump to a new ‘equilibrium’ state due to its sensitivity to past radiation forcing (Hansen *et al*., 1984), or did its systems cross a threshold (Barnosky *et al*., 2012) possibly linked to the rise in the concentration of atmospheric CO_2_? Our results based on CMIP5 and statistical modelling indicate that a combination of warming from natural and anthropogenic forcing was responsible as a rebound from the cooling that followed the El Chichón eruption together with a reduction in stratospheric aerosols, although the relative contribution of each forcing factor is still unclear. While the present generation of climate models can reproduce the general features of global temperature change and the cooling that follows major volcanic eruptions (IPCC Chap. 9, 2013), up to now they have not been able to replicate observed regime shifts (Lo & Hsu, 2010) or simulate the dynamic response to external forcings that includes the effects of volcanism (Driscoll *et al*., 2012).

If the above‐combined anthropogenic and natural forcing thesis is correct, why was there no equivalent regime change after the larger Pinatubo eruption? Changes in direct and diffuse radiation as measured at Mauna Loa (Robock, 2000) were much greater for El Chichón in 1982 than for the eruption of Mount Pinatubo almost a decade later (Fig. S5). The recovery from El Chichón was also more rapid, and the aerosol plume initially covered a greater area than for the following Pinatubo eruption (to 30°N compared to 15°N) (Robock, 2000), which favours a stronger shift impulse from the former. This fits with measurements that show that much of the aerosol plume from Pinatubo moved rapidly to the south of the equator after the eruption whereas most of the El Chichón plume remained in the Northern Hemisphere (McCormick *et al*., 1995). The seasonal timing of the eruptions (figure 7 in Post *et al*., 1996) and differences in stratospheric wind direction and strength, linked to the quasi‐biennial oscillation (McCormick *et al*., 1995), have also contributed.

A reversal in trend from global dimming to global brightening, with stronger surface solar radiation from the late 1980s, preceded and possibly masked some of the effects of the Pinatubo eruption. The enhanced radiation has been attributed to a substantial reduction in anthropogenic sulphur aerosols (Wild, 2009) that occurred at this time (Stern, 2006). Parallel and likely associated changes occurred in precipitation, reflecting a more active global hydrological cycle (Wild, 2009).

In the last decade, there has been an increasing debate on the possible need to deliberately geoengineer the climate to compensate for greenhouse gas induced global warming by either removing CO_2_ from the atmosphere or by reducing solar irradiance (Shepherd *et al*., 2009). Our results have considerable relevance to this debate. The speed, scale and global extent of the changes that took place in the 1980s have not been recognized until now, and therefore could not have been taken into account by geoengineering proposals. Current understanding that major volcanic eruptions only cause short‐term cooling of the Earth (Cole‐Dai, 2010) is contradicted by our demonstration of a longer‐term warming effect that involves the interaction of major volcanism with global warming. The cascading effects of the 1980s regime shift emphasize the vulnerability of the Earth to large scale human climate intervention.

The importance of the 1980s regime shift is shown here to have been unparalleled within at least the last century. It has been little recognized in the past due to a paucity of long‐term time series that are maintained and sampled in the same consistent way over decades, and a compartmentalization of science with insufficient communication between different disciplines. Many factors related to atmospheric and oceanic circulation, volcanism, latent and sensible heat transport, cloudiness, aerosol effects, and shortwave and longwave radiation are likely to have been involved. Given the scale and global extent of the shift, the public, policy makers and the scientific community need to be made more aware of the importance of such events. We need to improve our ability to forecast and model the occurrence, magnitude and consequences of regime shifts and include their effects in risk assessments for proposed geoengineering approaches to modify the climate. The enormous impact of the regime shift is seen especially in the land and ocean carbon sinks; a key issue for humanity is how these ecosystem services will behave in the future. The wide range of changes associated with the 1980s regime shift supports a threshold thesis that moved the whole global system into a new, rapidly warming state, with compounding consequences.

## Supporting information


**Figure S1**. Autocorrelograms (a) for observed and (b) for simulated time series.

**Figure S2**. Comparison of multiple STARS on real and artificial time series: shift years.

**Figure S3**. Comparison of multiple STARS on real and artificial time series: shift strength.

**Figure S4**. Replotted figure SPM 1a from IPCC Summary for Policy Makers (2013).

**Figure S5**. Effects of the El Chichón and Pinatubo eruptions on radiation, redrawn from Robock (2000).

**Table S1**. Excel Database that includes all the annual values of the 72 analysed time series.

**Table S2**. Supporting source, background and methodology citations.

**Table S3**. Additional information and notes on the time series presented in Fig. 2.

**Table S4**. Coordinates for six regions of tropical hurricanes/storms in Fig. 6.

**Table S5**. Shift year of the time series from Fig. 2 included in the regions of Fig. 6. A compendium of all the supplementary figures and Tables 2‐5 with their legends plus additional references for Table S2.
