# Supplementary figures and images for: Global impacts of the 1980s regime shift
Source: Glob Chang Biol. 2015 Nov 23;22(2):682–703. doi: 10.1111/gcb.13106 (PMC4738433; doi:10.1111/gcb.13106)

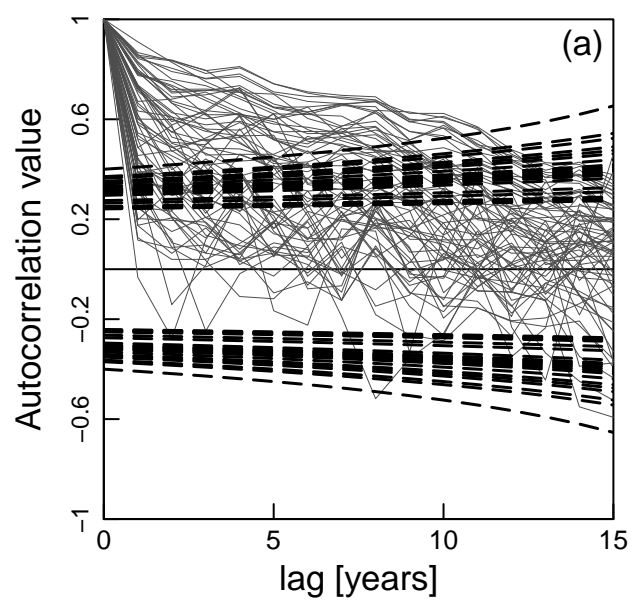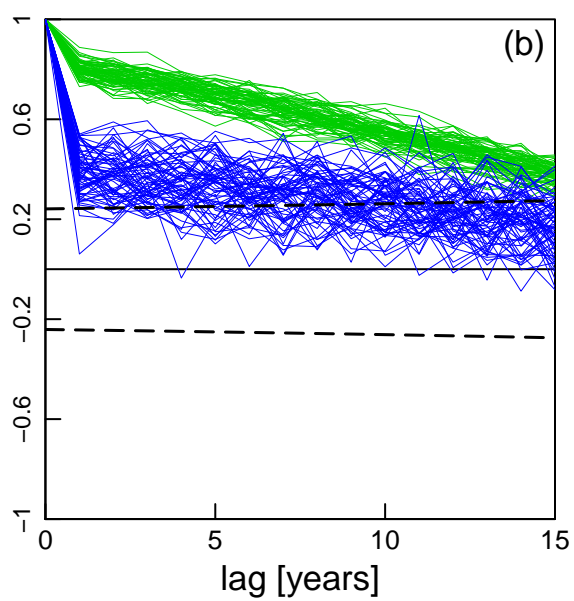

Supplement: Supplementary file 1 — Figure S1. Autocorrelograms (a) for observed and (b) for simulated time series. [file GCB-22-682-s001.pdf]

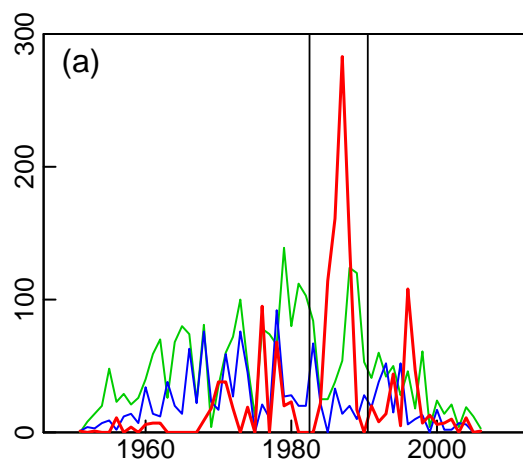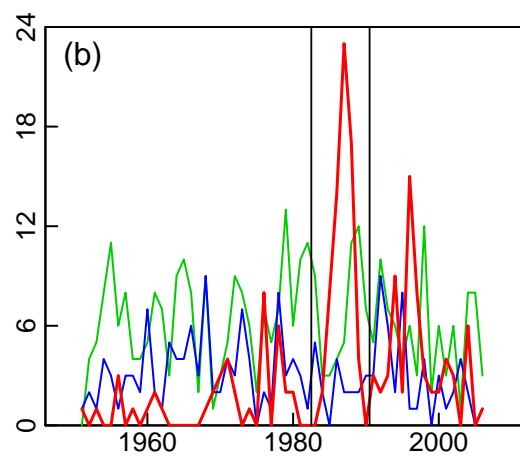

Supplement: Supplementary file 2 — Figure S2. Comparison of multiple STARS on real and artificial time series: shift years. [file GCB-22-682-s002.pdf]

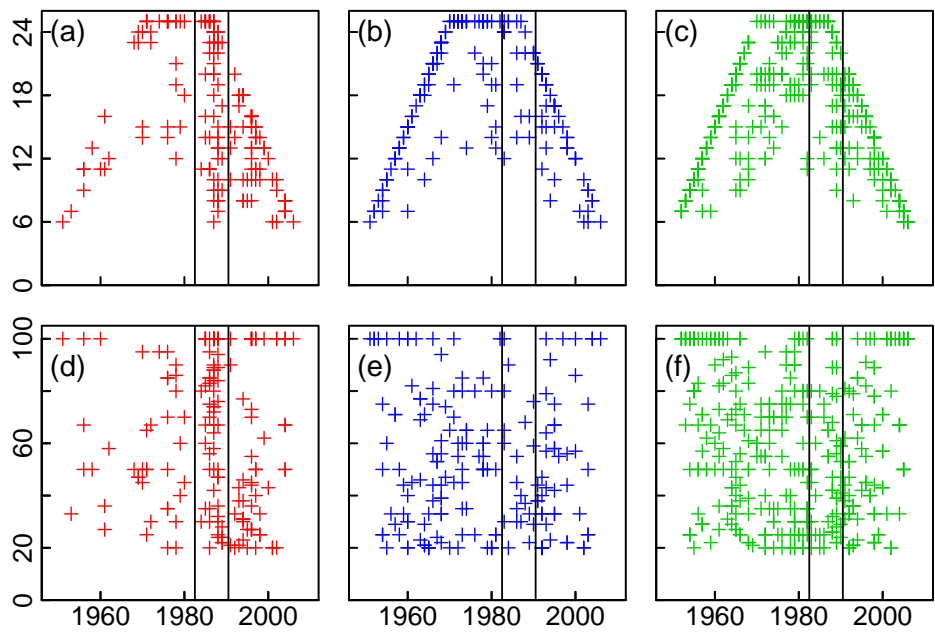

Supplement: Supplementary file 3 — Figure S3. Comparison of multiple STARS on real and artificial time series: shift strength. [file GCB-22-682-s003.pdf]

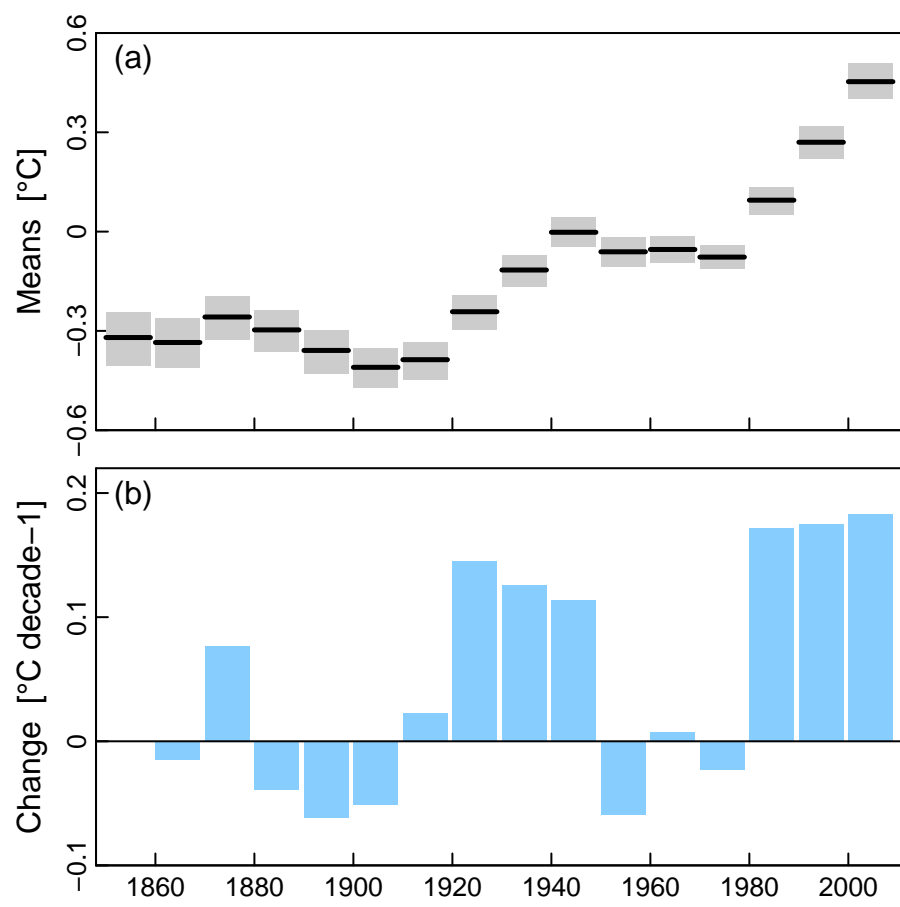

Supplement: Supplementary file 4 — Figure S4. Replotted figure SPM 1a from IPCC Summary for Policy Makers (2013). [file GCB-22-682-s004.pdf]

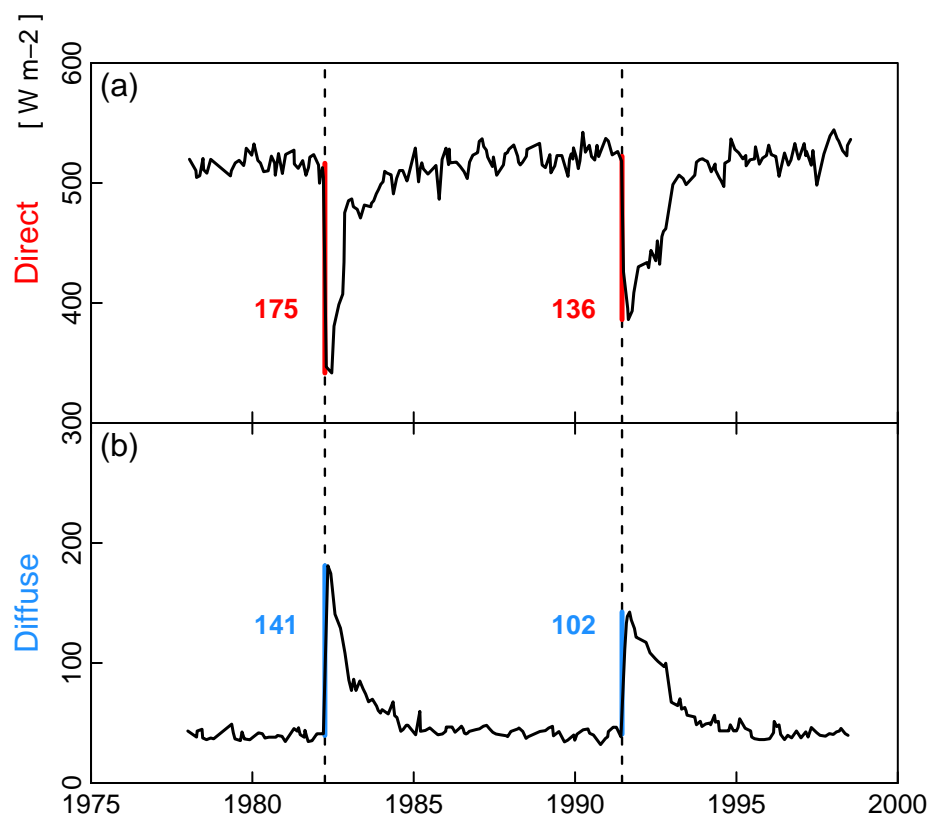

Supplement: Supplementary file 5 — Figure S5. Effects of the El Chichón and Pinatubo eruptions on radiation, redrawn from Robock (2000). [file GCB-22-682-s005.pdf]
