# Supplementary material for: Global impacts of the 1980s regime shift
Source: Glob Chang Biol. 2015 Nov 23;22(2):682–703. doi: 10.1111/gcb.13106 (PMC4738433; doi:10.1111/gcb.13106)
Supplement: Supplementary file 7 — Table S2. Supporting source, background and methodology citations. [file GCB-22-682-s007.docx]

**Table S2**

| **Graph title** | | **Supporting citations** |  | **Graph title** | | **Supporting citations** |
| --- | --- | --- | --- | --- | --- | --- |
| **Fig. 2** | | | | | | |
| **a** | Swiss ~26 km stratospheric temperature | Brocard *et al.*, 2013 |  | **w** | Western USA Wildfire duration (days) | Westerling *et al.*, 2006 |
| **b** | Swiss ~5 km tropospheric temperature | Brocard *et al.*, 2013 |  | **x** | NH satellite vegetation | Myneni *et al.*, 1997 |
| **c** | Meridional wind speed 60-75°N ~5 km a.s.l. | Xiao *et al.*, 2012 |  | **y** | NH start of thermal growing season | Barichivich *et al.*, 2013; Barichivich *et al.*, 2012 |
| **d** | Zonal wind speed 60-75°N ~5 km a.s.l. | Xiao *et al.*, 2012 |  | **z** | NH length of thermal growing season | Barichivich *et al.*, 2013 |
| **e** | China spring dust storm frequency | Ding *et al.*, 2005 |  | **aa** | NH end of thermal growing season | Barichivich *et al.*, 2013; Barichivich *et al*., 2012 |
| **f** | Global tropical hurricane/storm days | Webster *et al.*, 2005 |  | **ab** | Japan Kyoto cherry blossom | Aono and Kazui, 2008 |
| **g** | NH seasonally varying Index (SV-NAM) | Ogi *et al.*, 2004 |  | **ac** | UK sand martin arrival | Sparks and Tryjanowski, 2007 |
| **h** | Arctic Oscillation Index (AO) | Thompson and Wallace, 1998 |  | **ad** | Germany grape vine ripening date | Bock *et al.*, 2011 |
| **i** | North Atlantic Oscillation (NAO DJFM) | Hurrell, 1995 |  | **ae** | Baltic river Daugava winter flow | Klavins *et al.*, 2009 |
| **j** | Arctic temperature (SST and LST) | Hansen *et al.*, 2006 |  | **af** | Switzerland river temperature | Hari *et al.*, 2006; Jakob *et al.*, 2002 |
| **k** | Arctic sea level pressure | Compo *et al.*, 2011 |  | **ag** | Switzerland river pH | Jakob *et al.*, 2002; Sigg and Stumm, 2011 |
| **l** | Alaska atmospheric CO_2_, Apr-Sep | Barichivich *et al.*, 2012; Thoning *et al.*, 1989 |  | **ah** | North Sea phytoplankton biomass | Reid *et al.*, 1998; Raitsos *et al.*, 2014 |
| **m** | Alaska atmospheric CO_2_, Oct-Mar | Barichivich *et al.*, 2012; Thoning *et al.*, 1989 |  | **ai** | North Sea temperature | Ingleby and Huddleston, 2007 |
| **n** | Global CO_2_ net land uptake | Beaulieu *et al.*, 2012; Sarmiento *et al.*, 2010 |  | **aj** | North Sea Skagerrak 50 m depth salinity | Danielssen *et al.*, 1996 |
| **o** | NH time-integrated temp. of thermal growing season | Barichivich *et al.*, 2013 |  | **ak** | Japan Sea temperature at 50 m depth | Tian *et al.*, 2008 |
| **p** | Western Antarctica temperature (LST) | Bromwich *et al.*, 2013 |  | **al** | North Pacific Kuroshio current flow | Japan Meteorological Agency 2006 |
| **q** | Western Antarctica sea-ice extent | Parkinson and Cavalieri, 2008 |  | **am** | Japan Sea deep living fish (egg numbers) | Fujino *et al.*, 2013 |
| **r** | NH spring snow extent | Brown and Robinson, 2011 |  | **an** | Japan Sea tuna catch | Tian *et al*., 2008 |
| **s** | Switzerland snow days,  Dec-Mar | Marty 2008 |  | **ao** | Germany lake algal spring bloom | Gerten and Adrian, 2000 |
| **t** | Baltic Sea sea-ice extent | Axell and Lindquist, 2005 |  | **ap** | Switzerland groundwater temperature | Figura *et al.*, 2011 |
| **u** | Arctic sea-ice volume (Sep) | Lindsay *et al.*, 2009 |  | **aq** | Switzerland Lake Zürich temperature | North *et al.,* 2013 |
| **v** | NH sea-ice extent (Sep) | Rayner *et al.*, 2003 |  | **ar** | SH annular Mode Index (SAM) | Marshall, 2003 |
| **Fig. 3** | | | | | | |
| **a** | Japan Kyoto cherry blossom blooming | Aono and Kazui, 2008 |  |  |  |  |
| **b** | Switzerland Liestal cherry blossom | Defila and Clot, 2001 |  |  |  |  |
| **c** | USA Washington D.C. cherry blossom | Chung *et al.*, 2011 |  |  |  |  |
| **Fig. 4** | | | | | | |
|  | Continents GHCN v3 | Lawrimore *et al.,* 2011 |  |  |  |  |
|  | Oceans HadSST3 | Kennedy *et al.,* 2011 |  |  |  |  |
| **Fig. 5** | | | | | | |
| **a-c** | GHCN v3 | Lawrimore *et al.,* 2011 |  | **g-i** | HadSST3 | Kennedy *et al.*, 2011 |
| **d-f** | CRUTEM4 | Jones *et al.,* 2012 |  | **j-l** | HadCRUT4 | Morice *et al.*, 2012 |
