# Supplementary material for: Global impacts of the 1980s regime shift
Source: Glob Chang Biol. 2015 Nov 23;22(2):682–703. doi: 10.1111/gcb.13106 (PMC4738433; doi:10.1111/gcb.13106)
Supplement: Supplementary file 8 — Table S3. Additional information and notes on the time series presented in Fig. 2. [file GCB-22-682-s008.docx]

**Table S3.** Additional information and notes on the time series presented in Fig. 2.

Dimensionless time series (i. e. NDVI, salinity, pH and indices) have no units; this is indicated with a dash ‘-’.

|  | **Time series title** | |  |  |  |  |
| --- | --- | --- | --- | --- | --- | --- |
|  | **1** | **Processed by / Data Originator / Source** |  |  |  |  |
|  | **2** | **Organisation** |  |  |  |  |
|  | **3** | **Notes** | **Latitude / Longitude** | **Time period** | **Units:** | **Shift years** |
| **ATMOSPHERE** | | | | | | |
| **a** | **Swiss ~26 km stratospheric temperature** | | *46.82°N, 6.95°E* | 1976-2011 | **°C** | **1986** |
| **b** | **Swiss ~5 km tropospheric temperature** | | *46.82°N, 6.95°E* | 1976-2011 | **°C** | **1988** |
|  | 1,2 | René Stübi, from Swiss Federal Office of Meteorology and Climatology (MeteoSwiss), Payerne, Switzerland | | | | |
| **c** | **Meridional wind speed 60-75°N ~5 km a.s.l.** | | *360° circumglobal band 60°N-75°N* | 1948-2010 | **m s^-1^** | **1956, 1988** |
| **d** | **Zonal wind speed 60-75°N ~5 km a.s.l.** | | *360° circumglobal band 60°N-75°N* | 1948-2010 | **m s^-1^** | **1988, 1997** |
|  | 1,2 | Dong Xiao, from Chinese Academy of Meteorological Sciences, Beijing, China | | | | |
| **e** | **China spring dust storm frequency** | | *35°N-48°N, 75°E-102°E* | 1960-2011 | **No. of days of dust storms** | **1984** |
|  | 1 | Ruiqiang Ding |  |  |  |  |
|  | 2 | State Key Laboratory of Numerical Modeling for Atmospheric Sciences and Geophysical Fluid Dynamics (LASG), Beijing, China | | | | |
| **f** | **Global tropical hurricane/storm (d)** | | *See Supporting table S4* | 1970-2004 | **No. of days per year** | **1988** |
|  | 1 | Peter J. Webster, Violeta E. Toma & Hai-Ru Chang | |  |  |  |
|  | 2 | Georgia Institute of Technology, Atlanta, USA | | | | |
|  | 3 | Global total derived from six-hourly reports summed from six ocean basins. | | | | |
| **g** | **NH SV−NAM Index** | | *> 40°N* | 1948-2009 | **­-** | **1987, 1994** |
|  | 1 | http://wwwoa.ees.hokudai.ac.jp/people/yamazaki/SV-NAM/index.html | | | | |
|  | 2 | Hokkaido University, Japan | | | | |
|  | 3 | The index is the standardised score of the leading empirical orthogonal function (EOF) of the monthly and zonally averaged geopotential height fields poleward of 40°N from 1000 hPa to 200 hPa. | | | | |
| **h** | **Arctic Oscillation Index (AO)** | | *> 20°N* | 1950-2012 | **­-** | **1987, 1988, 1994** |
|  | 1 | http://www.esrl.noaa.gov/psd/data/climateindices/list/#AO | | | | |
|  | 2 | NOAA Earth System Research Laboratory, Boulder, USA | | | | |
|  | 3 | Determined as the leading orthogonal empirical function at the height of the 1000 hPa surface (approximately sea level pressure) and plotted as anomalies relative to 1979-2000. | | | | |
| **i** | **North Atlantic Oscillation (NAO, DJFM)** | | *38.71-65.07°N, 9.14-22.72°W* | 1946-2011 | **hPa** | **1961, 1971, 1972,** |
|  | 1 | https://climatedataguide.ucar.edu/sites/default/files/climate_index_files/nao_station_djfm.ascii | | | | **1995** |
|  | 2 | Climate Analysis Section, NCAR, Boulder, USA | | | | |
|  | 3 | The winter (December-March) NAO index used here is the normalised sea level pressure (SLP) between Lisbon, Portugal and Stykkisholmur/Reykjavik as anomalies relative to 1864-1983. | | | | |
| **j** | **Arctic temperature (SST and LST)** | | *> ~64°N* | 1946-2012 | **°C** | **1987, 1999, 2004** |
|  | 1 | GISS Land-Ocean Temperature Index (LOTI), http://data.giss.nasa.gov/gistemp/tabledata_v3/ZonAnn.Ts+dSST.txt | | | | |
|  | 2 | NASA Goddard Institute for Space Studies (GISS), New York, USA | | | | |
| **k** | **Arctic sea level pressure** | | *> 70°N* | 1946-2010 | **hPa** | **1987, 1995** |
|  | 1 | <http://www.esrl.noaa.gov/psd/data/gridded/data.20thC_ReanV2.monolevel.mm.html> | | | | |
|  | 2 | NOAA Earth System Research Laboratory, Boulder, USA | |  |  |  |
| **l** | **Alaska atmospheric CO_2_, Apr−Sep** | | *71.32°N, 156.61°W* | 1972-2010 | **ppm** | **1985, 1988** |
| **m** | **Alaska atmospheric CO_2_, Oct−Mar** | | *71.32°N, 156.61°W* | 1972-2010 | **ppm** | **1986, 1988** |
|  | 1 | Jonathan Barichivich and Renata E. Hari; <ftp://ftp.cmdl.noaa.gov/ccg/co2/GLOBALVIEW> | | | | |
|  | 2 | Cooperative Atmospheric Data Integration Project – CarbonDioxide, Boulder, USA | | | | |
| **n** | **Global CO_2_ net land uptake** | | *90°S-90°N* | 1966-2003 | **Pg C year^-1^** | **1988** |
|  | 1,2 | Claudie Beaulieu, from Ocean and Earth Science, University of Southampton, UK | | | | |
|  | 3 | The net land uptake (NLU) is calculated as a difference between annually reported global fossil fuel emissions, the growth rate of atmospheric CO_2_ as a mean of measurements at Mauna Loa and the South Pole and the ocean uptake as a mean calculated from four ocean biogeochemical models. The NLU, as opposed to the land uptake, does not require specification of land use sources, which have remained approximately constant from 1959 to 2006. | | | | |
| **o** | **NH integrated temperature growing season** | | *> 35°N* | 1950-2011 | **°C** | **1987, 2004** |
|  | 1,2 | Jonathan Barichivich, from University of East Anglia, UK | | | | |
|  | 3 | The thermal growing season is defined as the period of the year with daily mean air temperatures > 5°C. Plotted as anomalies relative to 1961-1990. | | | |  |
| **p** | **Western Antarctica temperature (LST)** | | *80°S, 120°W* | 1957-2012 | **°C** | **1986** |
|  | 1 | David H. Bromwich, http://polarmet.osu.edu/Byrd_recon/ | |  |  |  |
|  | 2 | The Ohio State University, Columbus, USA | |  |  |  |

|  | **Time series title** | |  |  |  |  |
| --- | --- | --- | --- | --- | --- | --- |
|  | **1** | **Processed by / Data Originator / Source** |  |  |  |  |
|  | **2** | **Organisation** |  |  |  |  |
|  | **3** | **Notes** | **Latitude / Longitude** | **Time period** | **Units:** | **Shift years** |
| **CRYOSPHERE** | | | | | | |
| **q** | **Western Antarctica sea−ice extent** | | *50°S-75°S, 60°W-130°W* | 1979-2010 | **10^6^ km^2^** | **1987** |
|  | 1 | Claire L. Parkinson | *or coast of Antarctica* |  |  |  |
|  | 2 | NASA Goddard Space Flight Center, Greenbelt, USA | |  |  |  |
| **r** | **NH spring snow extent** | | *0-90°N* | 1946-2010 | **10^6^ km^2^** | **1953, 1987** |
|  | 1 | Ross Brown, see also: http://www.the-cryosphere.net/5/219/2011/tc-5-219-2011-supplement.zip | | | | |
|  | 2 | Climate Research Division, Environment Canada, Montreal, Canada | | | | |
| **s** | **Swiss snow days, Dec−Mar** | | *45.82°N-47.81°N, 5.96°E-10.49°E* | 1946 to 2012 | **snow days** | **1987** |
|  | 1,2 | Christoph Marty, from WSL Institute for Snow and Avalanche Research, Davos, Switzerland and the Swiss Federal Office of Meteorology and Climatology (MeteoSwiss) | | | | |
|  |  |  |  |  |  |  |
|  | 3 | Mean of seven low-altitude stations (201–800 m). For this altitudinal band a snow day is when the snow depth exceeds a threshold of 5 cm. | | | | |
| **t** | **Baltic Sea sea−ice extent** | | *53.91°N-65.91°N, 9.43°E-30.3°E, and Kattegat up to the tip of Skagen at 57.75°N* | 1946-2012 | **10^6^ km^2^** | **1987** |
|  |  |  |  |  |  |  |
|  | 1 | Lars B. Axell and Karin Borenäs |  |  |  |  |
|  | 2 | Sveriges Meteorologiska och Hydrologiska Institut (SMHI), Norrköping and Gothenburg, Sweden | | | | |
|  | 3 | From 1957 to 2012 the time series is based on digitized hand-drawn ice charts and prior to 1957 on a least-squares method applied to observations along the Swedish coast. The total area evaluated covers 420∙10^3^ km^2^ with the maximum ice extent due to the methodology prior to 1957 equal to ~351∙10^3^ km^2^. | | | | |
| **u** | **Arctic sea−ice volume (Sep)** | | *> 65°N* | 1950-2012 | **10^3^ km^3^** | **1960, 1980, 1988,  1992, 1994, 1997,**  **2004** |
|  | 1 | Ron Lindsay, from the PIOMAS ice-ocean coupled model | |  |  |  |
|  | 2 | Polar Science Center, University of Washington | |  |  |  |
| **v** | **NH sea−ice extent (Sep)** | | *0-90°N* | 1960-2011 | **10^3^ km^2^** | **1978, 1989, 1998,** |
|  | 1 | Ron Lindsay, from the Hadley Centre Global Sea Ice and Sea Surface Temperature (HadISST1)  gridded dataset http://www.metoffice.gov.uk/hadobs/hadisst/ | | | | **2001, 2004** |
|  | 2 | Polar Science Center, University of Washington, Seattle, USA | | | | |
| **TERRESTRIAL** | | | | | | |
| **w** | **Western USA Wildfire duration (d)** | | *31°N-49°N, 102°W-125°W* | 1970-2003 | **No. of days per fire** | **1985** |
|  | 1 | Anthony L. Westerling |  |  |  |  |
|  | 2 | University of California, Merced, USA |  |  |  |  |
|  | 3 | The time series is based on 1166 large (> 400 ha) forest wildfires. | |  |  |  |
| **x** | **NH satellite vegetation** | | *> 45°N* | 1982-2010 | **­-** | **1987, 1988, 1996** |
|  | 1 | NDVI3g (third generation Global Inventory Modeling and Mapping System (GIMMS) NDVI | | |  |  |
| **y** | **NH start thermal growing season** | | *> 35°N* | 1950-2011 | **day of the yr** | **1972, 1988** |
| **z** | **NH length thermal growing season** | | *> 35°N* | 1950-2011 | **days** | **1987, 2004** |
| **aa** | **NH end thermal growing season** | | *> 35°N* | 1950-2011 | **day of the yr** | **1993, 2002** |
|  | 1 | Jonathan Barichivich |  |  |  |  |
|  | 2 | University of East Anglia, UK |  |  |  |  |
|  | 3 | The thermal growing season is defined as the period of the year with daily mean air temperatures > 5°C. Plotted as anomalies relative to 1961-1990. | | | | |
| **ab** | **Japan Kyoto cherry blossom** | | *35°N, 136.67°E* | 1946-2012 | **day of the yr** | **1988** |
|  | 1 | Yasuyuki Aono, data updated and revised | |  |  |  |
|  | 2 | Osaka Prefecture University, Japan | | | | |
| **ac** | **UK sand martin arrival** | | *50.72°N-54.19°N, 2.58°W-1.75°E* | 1950-2005 | **day of the yr** | **1976, 1980, 1988** |
|  | 1 | Tim Sparks |  |  |  |  |
|  | 2 | Coventry University, UK |  |  |  |  |
| **ad** | **Germany grape vine ripening date** | | *49.83° N, 9.87° E* | 1968-2010 | **day of the yr** | **1987, 1991** |
|  | 1 | Anna Bock |  |  |  |  |
|  | 2 | Technische Universität München, Freising, Germany | |  |  |  |
|  | 3 | Harvested from the vineyards of the Landesanstalt für Weinbau und Gartenbau (the regional office for viticulture and horticulture) Veitshöchheim, Franconia, Germany. | | | | |

|  | **Time series title** | | | **Latitude / Longitude** | **Time period** | **Units:** | **Shift years** |
| --- | --- | --- | --- | --- | --- | --- | --- |
| **HYDROSPHERE (OCEAN AND FRESHWATER)** | | | | | | | |
| **ae** | **Baltic river Daugava winter flow** | | *55.2°N-57.4°N, 24°E-28.2°E* | | 1946-2010 | **m^3^ s^-1^** | **1987** |
|  | 1 | Maris Klavins |  | |  |  |  |
|  | 2 | University of Latvia, Riga, Latvia |  | |  |  |  |
|  | 3 | Basin area: 64'500 km^2^. |  | |  |  |  |
| **af** | **Swiss river temperature** | | *45.82°N-47.81°N, 5.9°E-10.49°E* | | 1978-2011 | **°C** | **1987** |
|  | 1 | Renata E. Hari |  | |  |  |  |
|  | 2 | Swiss Federal Office for the Environment (BAFU), Hydrology Division | | |  |  |  |
| **ag** | **Swiss river pH** | | *45.82°N-47.81°N, 5.9°E-10.49°E* | | 1977-2010 | **-** | **1991** |
|  | 1 | Renata E. Hari |  | |  |  |  |
|  | 2 | Swiss Federal Office for the Environment (BAFU), Hydrology Division | | | | | |
|  | 3 | Legislation to reduce phosphate inputs to lakes and rivers was introduced in Switzerland in 1986. This would have had the opposite effect to the observed increase in pH: less phosphate → less algal growth → more CO_2_ →lower pH. It is more likely that the higher pH reflects increased algal growth due to higher temperatures, more sunshine and a higher CO_2_ concentration or increased weathering. | | | | | |
| **ah** | **North Sea phytoplankton biomass** | | *51°N-61°N, 3°W-10°E* | | 1946-2011 | **Colour categories** | **1951, 1985** |
|  | 1 | Sir Alister Hardy Foundation for Ocean Science (SAHFOS). | | | |  |  |
|  | 2 | SAHFOS, Plymouth, UK | | | |  |  |
|  | 3 | Unit details: Four colour categories calibrated by acetone extracts and fluorescence | | | | | |
| **ai** | **North Sea temperature** | | *50°N-61°N and 3°W-9°E* | | 1950-2011 | **°C** | **1987, 2001** |
|  | 1 | Simon A. Good |  | |  |  |  |
|  | 2 | UK Met Office Hadley Centre, Exeter; EN3: quality controlled subsurface ocean temperature and salinity dataset. See: http://www.metoffice.gov.uk/hadobs/en3/ | | | | | |
| **aj** | **North Sea 50 m depth salinity** | | *58.13°N, 9.18°E* | | 1965-2007 | **­-** | **1988** |
|  | 1 | Else Juul Green, http://ocean.ices.dk/HydChem/HydChem.aspx?plot=yes | | | | | |
|  | 2 | International Council for the Exploration of the Seas, Copenhagen, Denmark | | | | | |
|  | 3 | The sampling location is equivalent to the Norwegian station Z220, which is 20 miles from the Norwegian coast. Measurements have been taken approximately once a month. | | | | | |
| **ak** | **Japan Sea temperature at 50 m depth** | | *33-38°N, 130-136°E* | | 1964-2008 | **°C** | **1987** |
|  | 1 | Yongjun Tian from the Japan Sea National Fisheries Research Institute. | | | | | |
|  | 2 | Japan Sea National Fisheries Research Institute, Niigata, Japan. | | | | | |
|  | 3 | Monthly measurements taken in the Japan Sea. to cover the path of the Tsushima Current between Wakasa Bay in Kyoto Prefecture and Yamaguchi Prefecture, Japan and averaged for the area within 33-38°N, 130-136°E. | | | | | |
| **al** | **North Pacific Kuroshio current flow** | | *across 137°E between 3-34°N* | | 1972-2011 | **1 Sv =**  **10^6^ m^3^ s^-1^** | **1987** |
|  | 1 | Yongjun Tian from theJapan Meteorological Agency (JMA), Tokyo, Japan. | | | |  |  |
|  | 2 | Japan Sea National Fisheries Research Institute, Niigata, Japan. | | | | | |
|  | 3 | Current flow estimated from geostrophic calculations based on temperature and salinity profiles taken twice a year (summer = Jul-Sep and winter = Jan-Mar) on a standard north to south section during research cruises of the Japan Meteorological Agency (JMA). | | | | | |
| **am** | **Japan Sea deep living fish (eggs)** | | *between 34.45°N-41.17°N and* | | 1981-2005 | **No. of eggs per m^2^** | **1988** |
|  | 1 | Yongjun Tian from theJapan Meteorological Agency (JMA), Tokyo, Japan. | | | |  |  |
|  | 2 | Japan Sea National Fisheries Research Institute, Niigata, Japan. | | | | | |
|  | 3 | Sampling stations were located within approximately 185 km of the coast of Japan. This abundant mesopelagic (1000-100 m deep) species normally swims between 150 to 250 m during the daytime and migrates to shallower depths during the night. | | | | | |
| **an** | **Japan Sea tuna catch** | | *34°N-41.5°N, 131°E-141°E* | | 1964-2004 | **10^3^ tons** | **1991** |
|  | 1 | Yongjun Tian from theJapan Meteorological Agency (JMA), Tokyo, Japan. | | | |  |  |
|  | 2 | Japan Sea National Fisheries Research Institute, Niigata, Japan. | | | | | |
|  | 3 | Mostly comprising warm-water bluefin, albacore, and yellowfin tuna. | | | | | |
| **ao** | **Germany lake algal spring bloom** | | *13.65°E, 52.43°N* | | 1980-2010 | **Calendar week** | **1987** |
|  | 1 | Rita Adrian | | | |  |  |
|  | 2 | Leibniz- Institute of Freshwater Ecology and Inland Fisheries, Berlin, Germany. | | | | | |
|  | 3 | The timing refers to the calendar week of the year when maximum total phytoplankton biomass developed after ice-off. | | | | | |
| **ap** | **Swiss groundwater temperature** | | *Pump-stations: Kiesen 46.80°N, 7.57°E;Neuhausen 47.68°N, 8.61°E* | | 1970-2005 | **°C** | **1987** |
|  | 1 | Simon Figura |  |  |  |  |  |
|  | 2 | Energie Wasser Bern and Städtische Werke Schaffhausen und Neuhausen am Rheinfall | | | | | |
| **aq** | **Swiss Lake Zürich temperature** | | *47.37°N-47.20°N, 8.53°E-8.82°E* | | 1946-2005 | **°C** | **1987** |
|  | 1 | Ryan P. North. | | | |  |  |
|  | 2 | Oliver Köster from Wasserversorgung der Stadt Zürich | | | | | |
| **ar** | **SH Annular Mode Index (SAM)** | | *40°S and 65°S* | | 1957-2012 | **­-** | **1992, 1996** |
|  | 1 | http://www.nerc-bas.ac.uk/icd/gjma/sam.html | | | |  |  |
|  | 2 | British Antarctic Survey, Cambridge, UK | | | | | |
|  | 3 | The index used here is derived from a proxy zonal mean sea level pressure for 40°S and 65°S calculated from twelve meteorological stations that approximate to each of these latitudes, anomalies relative to 1971-2000. | | | | | |
