# Supplementary material for: Global impacts of the 1980s regime shift
Source: Glob Chang Biol. 2015 Nov 23;22(2):682–703. doi: 10.1111/gcb.13106 (PMC4738433; doi:10.1111/gcb.13106)
Supplement: Supplementary file 9 — Table S4. Coordinates for six regions of tropical hurricanes/storms in Fig. 6. [file GCB-22-682-s009.docx]

**Table S4**

| Ocean region | Minimum  latitude | Maximum  latitude | Minimum  longitude | Maximum  longitude |
| --- | --- | --- | --- | --- |
| A Eastern North Pacific | 5°N | 20°N | 90°W | 120°W |
| B North Atlantic | 5°N | 25°N | 20°W | 90°W |
| C North Indian | 5°N | 20°N | 55°E | 90°E |
| D South Indian | 5°S | 20°S | 50°E | 115°E |
| E Western North Pacific | 5°N | 20°N | 120°E | 180°E |
| F Southwest Pacific | 5°S | 20°S | 155°E | 180°E |
