# Supplementary material for: Global impacts of the 1980s regime shift
Source: Glob Chang Biol. 2015 Nov 23;22(2):682–703. doi: 10.1111/gcb.13106 (PMC4738433; doi:10.1111/gcb.13106)
Supplement: Supplementary file 10 — Table S5. Shift year of the time series from Fig. 2 included in the regions of Fig. 6. A compendium of all the supplementary figures and Tables 2‐5 with their legends plus additional references for Table S2. [file GCB-22-682-s010.docx]

**Table S5.** Shift year of the time series from Fig. 2 included in the regions of Fig. 6.

| **EUROPE INSET** | |  | |
| --- | --- | --- | --- |
| 2b | Switzerland Payerne ~5 km tropospheric air temperature, atmosphere | | 1988 |
| 2t | Baltic Sea sea-ice extent, cryosphere | | 1987 |
| 2ac | UK sand martin arrival date, terrestrial biosphere | | 1988 |
| 2ad | Germany grape vine ripening date, terrestrial biosphere | | 1987 |
| 2ae | Baltic river Daugava winter flow, terrestrial hydrosphere | | 1987 |
| 2af | Switzerland river temperature, terrestrial hydrosphere | | 1987 |
| 2ah | North Sea phytoplankton biomass, marine biosphere | | 1985 |
| 2ai | North Sea temperature, marine hydrosphere | | 1987 |
| 2ao | Germany Lake Müggelsee algal bloom spring timing, terrestrial biosphere | | 1987 |
| **JAPAN INSET** | | |  |
| 2ak | Japan Sea temperature at 50m depth, marine hydrosphere | | 1987 |
| 2al | Western North Pacific Kuroshio current flow, marine hydrosphere | | 1987 |
| 2ab | Japan Kyoto cherry blossom blooming, terrestrial biosphere | | 1988 |
| **GLOBAL MAP** | | |  |
| **North America** | | |  |
| 2m | Alaska Point Barrow atmospheric CO_2_ concentration (Apr-Sep), atmosphere | | 1985 |
| 2n | Alaska Point Barrow atmospheric CO_2_ concentration (Oct-Mar), atmosphere | | 1986 |
| 2w | Western USA Wildfire duration (days), terrestrial biosphere | | 1985 |
| **Asia** | | |  |
| 2e | China dust storm frequency (March-May), atmosphere | | 1984 |
| **Tropics** | | |  |
| 2f | Global tropical hurricane/storm days (mean areas A-F), atmosphere | | 1988 |
| **Antarctica** | | |  |
| 2p | Western Antarctica air surface temperature Byrd station, atmosphere | | 1986 |
| 2q | Western Antarctica sea-ice extent, cryosphere | | 1987 |
| **GLOBAL AND HEMISPHERIC TIME SERIES** | | |  |
| Arrows left to right: | | |  |
| 2n | Global CO_2_ net land uptake, atmosphere | | 1988 |
| 2r | Northern Hemisphere spring snow extent, cryosphere | | 1987 |
| 2x | Northern Hemisphere vegetation from satellites, terrestrial biosphere | | 1987 |
| 2j | Arctic combined sea and air surface temperature, atmosphere | | 1987 |
| 2k | Arctic sea level pressure, atmosphere | | 1987 |
| 2c | Meridional wind speed 60-75°N (360°) ~5 km above sea level, atmosphere | | 1988 |
| 2d | Zonal wind speed 60-75°N (360°) ~5 km above sea level, atmosphere | | 1988 |
