# Supplementary material for: Global impacts of the 1980s regime shift
Source: Glob Chang Biol. 2015 Nov 23;22(2):682–703. doi: 10.1111/gcb.13106 (PMC4738433; doi:10.1111/gcb.13106)
Supplement: Supplementary file 11 [file GCB-22-682-s011.docx]

**Supporting Information**


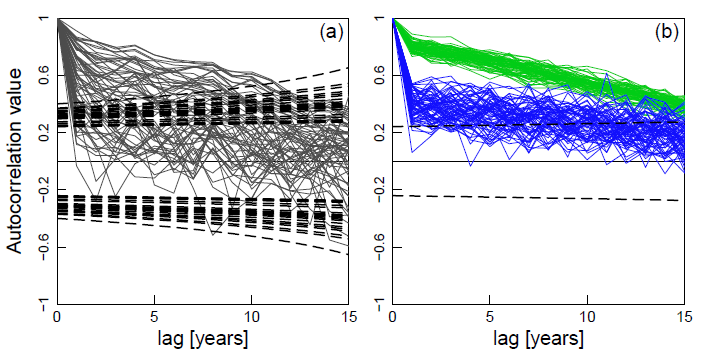


**Fig. S1**. Autocorrelograms (**a**) for observed and (**b**) for simulated time series: 70 highly autocorrelated time series (green) and 70 medium autocorrelated time series (blue) that correspond respectively to the maximum and medium autocorrelation observed in the real time series.


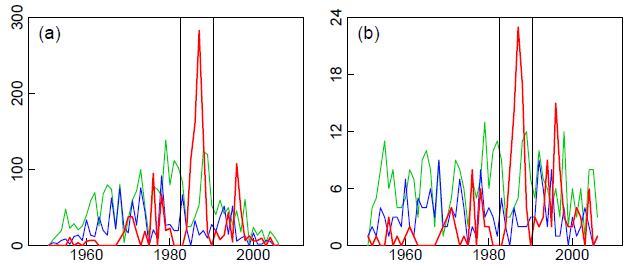


**Fig. S2**. Comparison of multiple STARS on real and artificial time series: shift years. Data plotted for 72 real time series (red), and 70 artificial time series (with medium – blue, and high autocorrelation - green) for the period from 1946-2012. The ‘period of interest’ (1983-1990) is marked by vertical lines. (**a**) Number of significant t-tests per year (p ≤ 0.05). (**b**) Number of significant shift years per year (p ≤ 0.05).


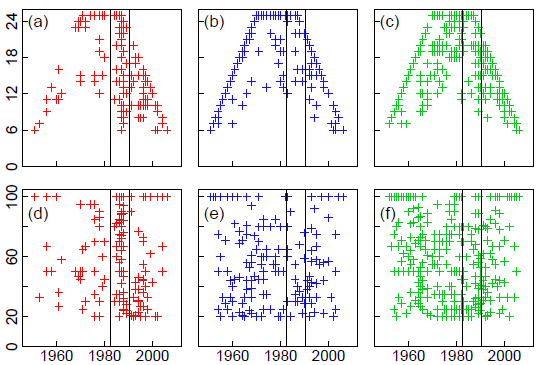


**Fig. S3**. Comparison of multiple STARS on real and artificial time series: shift strength. Real (**a**,**d**) and artificial (**b**,**c**,**e**,**f**) time series. Data and period analysed as in Supporting Information Fig. S2. (**a**-**c**) The length of the longest significant test-period for each shift year out of the 20 test-periods applied is between 6 to 25 years (y-axis). (**d-f**) The percentage of the number of significant t-tests (p ≤ 0.05) against the total number of possible t-tests for each shift year (y-axis). Results below 20% discarded.


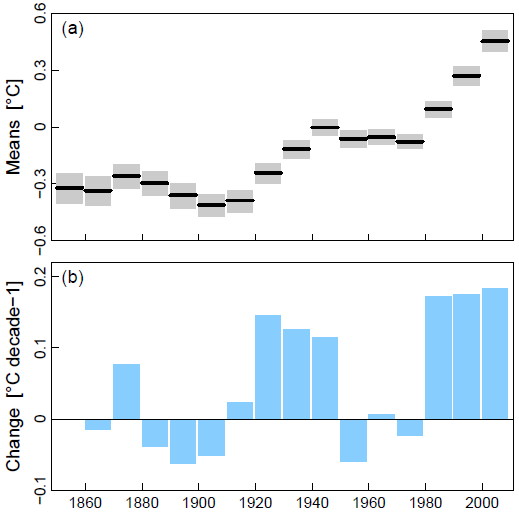


**Fig. S4**. Replotted figure SPM 1a from the IPCC Summary for Policy Makers (2013). Global temperature changes per decade: (**a**) Decadal global means of combined land and sea surface temperatures (HadCrut4.3.0.0) as anomalies relative to 1961-1990 for the period 1850 to 2009. Recalculated and re-plotted after figure SPM 1a in the IPCC Summary for Policy Makers (IPCC SPM, 2013); data courtesy Colin P. Mollis, Hadley Centre, Exeter, UK. (**b**) The change per decade.


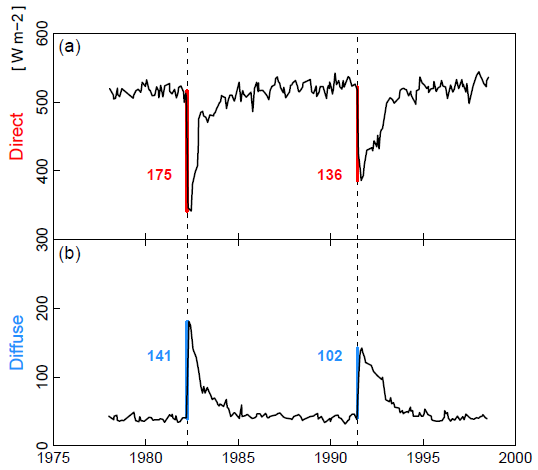


**Fig. S5**. Effects of the El Chichón and Pinatubo eruptions on radiation [W m^-2^].

Direct and diffuse broadband radiation measurements from the Mauna Loa Observatory against years. Redrawn from (Robock, 2000) with superimposed dashed lines for both volcanic eruptions showing the extent and its value as a number of (**a**) the reduction of direct (red) and (**b**) the enhancement of diffuse (blue) radiation immediately after the eruptions of El Chichón at the end of March 1982 and Pinatubo in June 1991 (dashed lines). The lower numbers for Pinatubo are 78% (direct) and 72% (diffuse) of the change seen after the earlier eruption. The data were obtained with a tracking pyrheliometer and shade disk pyranometer on mornings with clear skies at a solar zenith angle of 60°, equivalent to two relative air masses. Data from NOAA/ESRL/GMD/GRAD Radiation Archive: <ftp://aftp.cmdl.noaa.gov/data/radiation/baseline/> .

**Table S1**. Supporting Information Database

Excel file containing the data for all 72 time series analysed .

**Table S2**. Supporting source, background and methodology citations. The table addresses the time series presented in Figs. 2-5.

| **Graph title** | | **Supporting citations** |  | **Graph title** | | **Supporting citations** |
| --- | --- | --- | --- | --- | --- | --- |
| **Fig. 2** | | | | | | |
| **a** | Swiss ~26 km stratospheric temperature | Brocard *et al.*, 2013 |  | **w** | Western USA Wildfire duration (days) | Westerling *et al.*, 2006 |
| **b** | Swiss ~5 km tropospheric temperature | Brocard *et al.*, 2013 |  | **x** | NH satellite vegetation | Myneni *et al.*, 1997 |
| **c** | Meridional wind speed 60-75°N ~5 km a.s.l. | Xiao *et al.*, 2012 |  | **y** | NH start of thermal growing season | Barichivich *et al.*, 2013; Barichivich *et al.*, 2012 |
| **d** | Zonal wind speed 60-75°N ~5 km a.s.l. | Xiao *et al.*, 2012 |  | **z** | NH length of thermal growing season | Barichivich *et al.*, 2013 |
| **e** | China spring dust storm frequency | Ding *et al.*, 2005 |  | **aa** | NH end of thermal growing season | Barichivich *et al.*, 2013; Barichivich *et al*., 2012 |
| **f** | Global tropical hurricane/storm days | Webster *et al.*, 2005 |  | **ab** | Japan Kyoto cherry blossom | Aono and Kazui, 2008 |
| **g** | NH seasonally varying Index (SV-NAM) | Ogi *et al.*, 2004 |  | **ac** | UK sand martin arrival | Sparks and Tryjanowski, 2007 |
| **h** | Arctic Oscillation Index (AO) | Thompson and Wallace, 1998 |  | **ad** | Germany grape vine ripening date | Bock *et al.*, 2011 |
| **i** | North Atlantic Oscillation (NAO DJFM) | Hurrell, 1995 |  | **ae** | Baltic river Daugava winter flow | Klavins *et al.*, 2009 |
| **j** | Arctic temperature (SST and LST) | Hansen *et al.*, 2006 |  | **af** | Switzerland river temperature | Hari *et al.*, 2006; Jakob *et al.*, 2002 |
| **k** | Arctic sea level pressure | Compo *et al.*, 2011 |  | **ag** | Switzerland river pH | Jakob *et al.*, 2002; Sigg and Stumm, 2011 |
| **l** | Alaska atmospheric CO_2_, Apr-Sep | Barichivich *et al.*, 2012; Thoning *et al.*, 1989 |  | **ah** | North Sea phytoplankton biomass | Reid *et al.*, 1998; Raitsos *et al.*, 2014 |
| **m** | Alaska atmospheric CO_2_, Oct-Mar | Barichivich *et al.*, 2012; Thoning *et al.*, 1989 |  | **ai** | North Sea temperature | Ingleby and Huddleston, 2007 |
| **n** | Global CO_2_ net land uptake | Beaulieu *et al.*, 2012; Sarmiento *et al.*, 2010 |  | **aj** | North Sea Skagerrak 50 m depth salinity | Danielssen *et al.*, 1996 |
| **o** | NH time-integrated temp. of thermal growing season | Barichivich *et al.*, 2013 |  | **ak** | Japan Sea temperature at 50 m depth | Tian *et al.*, 2008 |
| **p** | Western Antarctica temperature (LST) | Bromwich *et al.*, 2013 |  | **al** | North Pacific Kuroshio current flow | Japan Meteorological Agency 2006 |
| **q** | Western Antarctica sea-ice extent | Parkinson and Cavalieri, 2008 |  | **am** | Japan Sea deep living fish (egg numbers) | Fujino *et al.*, 2013 |
| **r** | NH spring snow extent | Brown and Robinson, 2011 |  | **an** | Japan Sea tuna catch | Tian *et al*., 2008 |
| **s** | Switzerland snow days,  Dec-Mar | Marty 2008 |  | **ao** | Germany lake algal spring bloom | Gerten and Adrian, 2000 |
| **t** | Baltic Sea sea-ice extent | Axell and Lindquist, 2005 |  | **ap** | Switzerland groundwater temperature | Figura *et al.*, 2011 |
| **u** | Arctic sea-ice volume (Sep) | Lindsay *et al.*, 2009 |  | **aq** | Switzerland Lake Zürich temperature | North *et al.,* 2013 |
| **v** | NH sea-ice extent (Sep) | Rayner *et al.*, 2003 |  | **ar** | SH annular Mode Index (SAM) | Marshall, 2003 |
| **Fig. 3** | | | | | | |
| **a** | Japan Kyoto cherry blossom blooming | Aono and Kazui, 2008 |  |  |  |  |
| **b** | Switzerland Liestal cherry blossom | Defila and Clot, 2001 |  |  |  |  |
| **c** | USA Washington D.C. cherry blossom | Chung *et al.*, 2011 |  |  |  |  |
| **Fig. 4** | | | | | | |
|  | Continents GHCN v3 | Lawrimore *et al.,* 2011 |  |  |  |  |
|  | Oceans HadSST3 | Kennedy *et al.,* 2011 |  |  |  |  |
| **Fig. 5** | | | | | | |
| **a-c** | GHCN v3 | Lawrimore *et al.,* 2011 |  | **g-i** | HadSST3 | Kennedy *et al.*, 2011 |
| **d-f** | CRUTEM4 | Jones *et al.,* 2012 |  | **j-l** | HadCRUT4 | Morice *et al.*, 2012 |

**Table S3.** Additional information and notes on the time series presented in Fig. 2.

Dimensionless time series (i. e. NDVI, salinity, pH and indices) have no units; this is indicated with a dash ‘-’. Order as per Fig. 2: atmosphere, cryosphere, terrestrial, hydrophere (ocean and freshwater).

|  | **Time series title** | |  |  |  |  |
| --- | --- | --- | --- | --- | --- | --- |
|  | **1** | **Processed by / Data Originator / Source** |  |  |  |  |
|  | **2** | **Organisation** |  |  |  |  |
|  | **3** | **Notes** | **Latitude / Longitude** | **Time period** | **Units:** | **Shift years** |
| **ATMOSPHERE** | | | | | | |
| **a** | **Swiss ~26 km stratospheric temperature** | | *46.82°N, 6.95°E* | 1976-2011 | **°C** | **1986** |
| **b** | **Swiss ~5 km tropospheric temperature** | | *46.82°N, 6.95°E* | 1976-2011 | **°C** | **1988** |
|  | 1,2 | René Stübi, from Swiss Federal Office of Meteorology and Climatology (MeteoSwiss), Payerne, Switzerland | | | | |
| **c** | **Meridional wind speed 60-75°N ~5 km a.s.l.** | | *360° circumglobal band 60°N-75°N* | 1948-2010 | **m s^-1^** | **1956, 1988** |
| **d** | **Zonal wind speed 60-75°N ~5 km a.s.l.** | | *360° circumglobal band 60°N-75°N* | 1948-2010 | **m s^-1^** | **1988, 1997** |
|  | 1,2 | Dong Xiao, from Chinese Academy of Meteorological Sciences, Beijing, China | | | | |
| **e** | **China spring dust storm frequency** | | *35°N-48°N, 75°E-102°E* | 1960-2011 | **No. of days of dust storms** | **1984** |
|  | 1 | Ruiqiang Ding |  |  |  |  |
|  | 2 | State Key Laboratory of Numerical Modeling for Atmospheric Sciences and Geophysical Fluid Dynamics (LASG), Beijing, China | | | | |
| **f** | **Global tropical hurricane/storm days** | | *See Supp. Info. table S4* | 1970-2004 | **No. of days per year** | **1988** |
|  | 1 | Peter J. Webster, Violeta E. Toma & Hai-Ru Chang | |  |  |  |
|  | 2 | Georgia Institute of Technology, Atlanta, USA | | | | |
|  | 3 | Global total derived from six-hourly reports summed from six ocean basins. | | | | |
| **g** | **NH SV−NAM Index** | | *> 40°N* | 1948-2009 | **­-** | **1987, 1994** |
|  | 1 | http://wwwoa.ees.hokudai.ac.jp/people/yamazaki/SV-NAM/index.html | | | | |
|  | 2 | Hokkaido University, Japan | | | | |
|  | 3 | The index is the standardised score of the leading empirical orthogonal function (EOF) of the monthly and zonally averaged geopotential height fields poleward of 40°N from 1000 hPa to 200 hPa. | | | | |
| **h** | **Arctic Oscillation Index (AO)** | | *> 20°N* | 1950-2012 | **­-** | **1987, 1988, 1994** |
|  | 1 | http://www.esrl.noaa.gov/psd/data/climateindices/list/#AO | | | | |
|  | 2 | NOAA Earth System Research Laboratory, Boulder, USA | | | | |
|  | 3 | Determined as the leading orthogonal empirical function at the height of the 1000 hPa surface (approximately sea level pressure) and plotted as anomalies relative to 1979-2000. | | | | |
| **i** | **North Atlantic Oscillation (NAO, DJFM)** | | *38.71-65.07°N, 9.14-22.72°W* | 1946-2011 | **hPa** | **1961, 1971, 1972,** |
|  | 1 | https://climatedataguide.ucar.edu/sites/default/files/climate_index_files/nao_station_djfm.ascii | | | | **1995** |
|  | 2 | Climate Analysis Section, NCAR, Boulder, USA | | | | |
|  | 3 | The winter (December-March) NAO index used here is the normalised sea level pressure (SLP) between Lisbon, Portugal and Stykkisholmur/Reykjavik as anomalies relative to 1864-1983. | | | | |
| **j** | **Arctic temperature (SST and LST)** | | *> ~64°N* | 1946-2012 | **°C** | **1987, 1999, 2004** |
|  | 1 | GISS Land-Ocean Temperature Index (LOTI), http://data.giss.nasa.gov/gistemp/tabledata_v3/ZonAnn.Ts+dSST.txt | | | | |
|  | 2 | NASA Goddard Institute for Space Studies (GISS), New York, USA | | | | |
| **k** | **Arctic sea level pressure** | | *> 70°N* | 1946-2010 | **hPa** | **1987, 1995** |
|  | 1 | <http://www.esrl.noaa.gov/psd/data/gridded/data.20thC_ReanV2.monolevel.mm.html> | | | | |
|  | 2 | NOAA Earth System Research Laboratory, Boulder, USA | |  |  |  |
| **l** | **Alaska atmospheric CO_2_, Apr−Sep** | | *71.32°N, 156.61°W* | 1972-2010 | **ppm** | **1985, 1988** |
| **m** | **Alaska atmospheric CO_2_, Oct−Mar** | | *71.32°N, 156.61°W* | 1972-2010 | **ppm** | **1986, 1988** |
|  | 1 | Jonathan Barichivich and Renata E. Hari; <ftp://ftp.cmdl.noaa.gov/ccg/co2/GLOBALVIEW> | | | | |
|  | 2 | Cooperative Atmospheric Data Integration Project – CarbonDioxide, Boulder, USA | | | | |
| **n** | **Global CO_2_ net land uptake** | | *90°S-90°N* | 1966-2003 | **Pg C year^-1^** | **1988** |
|  | 1,2 | Claudie Beaulieu, from Ocean and Earth Science, University of Southampton, UK | | | | |
|  | 3 | The net land uptake (NLU) is calculated as a difference between annually reported global fossil fuel emissions, the growth rate of atmospheric CO_2_ as a mean of measurements at Mauna Loa and the South Pole and the ocean uptake as a mean calculated from four ocean biogeochemical models. The NLU, as opposed to the land uptake, does not require specification of land use sources, which have remained approximately constant from 1959 to 2006. | | | | |
| **o** | **NH integrated temperature growing season** | | *> 35°N* | 1950-2011 | **°C** | **1987, 2004** |
|  | 1,2 | Jonathan Barichivich, from University of East Anglia, UK | | | | |
|  | 3 | The thermal growing season is defined as the period of the year with daily mean air temperatures > 5°C. Plotted as anomalies relative to 1961-1990. | | | |  |
| **p** | **Western Antarctica temperature (LST)** | | *80°S, 120°W* | 1957-2012 | **°C** | **1986** |
|  | 1 | David H. Bromwich, http://polarmet.osu.edu/Byrd_recon/ | |  |  |  |
|  | 2 | The Ohio State University, Columbus, USA | |  |  |  |
|  | **Time series title** | |  |  |  |  |
|  | **1** | **Processed by / Data Originator / Source** |  |  |  |  |
|  | **2** | **Organisation** |  |  |  |  |
|  | **3** | **Notes** | **Latitude / Longitude** | **Time period** | **Units:** | **Shift years** |
| **CRYOSPHERE** | | | | | | |
| **q** | **Western Antarctica sea−ice extent** | | *50°S-75°S, 60°W-130°W* | 1979-2010 | **10^6^ km^2^** | **1987** |
|  | 1 | Claire L. Parkinson | *or coast of Antarctica* |  |  |  |
|  | 2 | NASA Goddard Space Flight Center, Greenbelt, USA | |  |  |  |
| **r** | **NH spring snow extent** | | *0-90°N* | 1946-2010 | **10^6^ km^2^** | **1953, 1987** |
|  | 1 | Ross Brown, see also: http://www.the-cryosphere.net/5/219/2011/tc-5-219-2011-supplement.zip | | | | |
|  | 2 | Climate Research Division, Environment Canada, Montreal, Canada | | | | |
| **s** | **Swiss snow days, Dec−Mar** | | *45.82°N-47.81°N, 5.96°E-10.49°E* | 1946 to 2012 | **snow days** | **1987** |
|  | 1,2 | Christoph Marty, from WSL Institute for Snow and Avalanche Research, Davos, Switzerland and the Swiss Federal Office of Meteorology and Climatology (MeteoSwiss) | | | | |
|  |  |  |  |  |  |  |
|  | 3 | Mean of seven low-altitude stations (201–800 m). For this altitudinal band a snow day is when the snow depth exceeds a threshold of 5 cm. | | | | |
| **t** | **Baltic Sea sea−ice extent** | | *53.91°N-65.91°N, 9.43°E-30.3°E, and Kattegat up to the tip of Skagen at 57.75°N* | 1946-2012 | **10^6^ km^2^** | **1987** |
|  |  |  |  |  |  |  |
|  | 1 | Lars B. Axell and Karin Borenäs |  |  |  |  |
|  | 2 | Sveriges Meteorologiska och Hydrologiska Institut (SMHI), Norrköping and Gothenburg, Sweden | | | | |
|  | 3 | From 1957 to 2012 the time series is based on digitized hand-drawn ice charts and prior to 1957 on a least-squares method applied to observations along the Swedish coast. The total area evaluated covers 420∙10^3^ km^2^ with the maximum ice extent due to the methodology prior to 1957 equal to ~351∙10^3^ km^2^. | | | | |
| **u** | **Arctic sea−ice volume (Sep)** | | *> 65°N* | 1950-2012 | **10^3^ km^3^** | **1960, 1980, 1988,  1992, 1994, 1997,**  **2004** |
|  | 1 | Ron Lindsay, from the PIOMAS ice-ocean coupled model | |  |  |  |
|  | 2 | Polar Science Center, University of Washington | |  |  |  |
| **v** | **NH sea−ice extent (Sep)** | | *0-90°N* | 1960-2011 | **10^3^ km^2^** | **1978, 1989, 1998,** |
|  | 1 | Ron Lindsay, from the Hadley Centre Global Sea Ice and Sea Surface Temperature (HadISST1)  gridded dataset http://www.metoffice.gov.uk/hadobs/hadisst/ | | | | **2001, 2004** |
|  | 2 | Polar Science Center, University of Washington, Seattle, USA | | | | |
| **TERRESTRIAL** | | | | | | |
| **w** | **Western USA Wildfire duration (d)** | | *31°N-49°N, 102°W-125°W* | 1970-2003 | **No. of days per fire** | **1985** |
|  | 1 | Anthony L. Westerling |  |  |  |  |
|  | 2 | University of California, Merced, USA |  |  |  |  |
|  | 3 | The time series is based on 1166 large (> 400 ha) forest wildfires. | |  |  |  |
| **x** | **NH satellite vegetation** | | *> 45°N* | 1982-2010 | **­-** | **1987, 1988, 1996** |
|  | 1 | NDVI3g (third generation Global Inventory Modeling and Mapping System (GIMMS) NDVI | | |  |  |
| **y** | **NH start thermal growing season** | | *> 35°N* | 1950-2011 | **day of the yr** | **1972, 1988** |
| **z** | **NH length thermal growing season** | | *> 35°N* | 1950-2011 | **days** | **1987, 2004** |
| **aa** | **NH end thermal growing season** | | *> 35°N* | 1950-2011 | **day of the yr** | **1993, 2002** |
|  | 1 | Jonathan Barichivich |  |  |  |  |
|  | 2 | University of East Anglia, UK |  |  |  |  |
|  | 3 | The thermal growing season is defined as the period of the year with daily mean air temperatures > 5°C. Plotted as anomalies relative to 1961-1990. | | | | |
| **ab** | **Japan Kyoto cherry blossom** | | *35°N, 136.67°E* | 1946-2012 | **day of the yr** | **1988** |
|  | 1 | Yasuyuki Aono, data updated and revised | |  |  |  |
|  | 2 | Osaka Prefecture University, Japan | | | | |
| **ac** | **UK sand martin arrival** | | *50.72°N-54.19°N, 2.58°W-1.75°E* | 1950-2005 | **day of the yr** | **1976, 1980, 1988** |
|  | 1 | Tim Sparks |  |  |  |  |
|  | 2 | Coventry University, UK |  |  |  |  |
| **ad** | **Germany grape vine ripening date** | | *49.83° N, 9.87° E* | 1968-2010 | **day of the yr** | **1987, 1991** |
|  | 1 | Anna Bock |  |  |  |  |
|  | 2 | Technische Universität München, Freising, Germany | |  |  |  |
|  | 3 | Harvested from the vineyards of the Landesanstalt für Weinbau und Gartenbau (the regional office for viticulture and horticulture) Veitshöchheim, Franconia, Germany. | | | | |

|  | **Time series title** | | | **Latitude / Longitude** | **Time period** | **Units:** | **Shift years** |
| --- | --- | --- | --- | --- | --- | --- | --- |
| **HYDROSPHERE (OCEAN AND FRESHWATER)** | | | | | | | |
| **ae** | **Baltic river Daugava winter flow** | | *55.2°N-57.4°N, 24°E-28.2°E* | | 1946-2010 | **m^3^ s^-1^** | **1987** |
|  | 1 | Maris Klavins |  | |  |  |  |
|  | 2 | University of Latvia, Riga, Latvia |  | |  |  |  |
|  | 3 | Basin area: 64'500 km^2^. |  | |  |  |  |
| **af** | **Swiss river temperature** | | *45.82°N-47.81°N, 5.9°E-10.49°E* | | 1978-2011 | **°C** | **1987** |
|  | 1 | Renata E. Hari |  | |  |  |  |
|  | 2 | Swiss Federal Office for the Environment (BAFU), Hydrology Division | | |  |  |  |
| **ag** | **Swiss river pH** | | *45.82°N-47.81°N, 5.9°E-10.49°E* | | 1977-2010 | **-** | **1991** |
|  | 1 | Renata E. Hari |  | |  |  |  |
|  | 2 | Swiss Federal Office for the Environment (BAFU), Hydrology Division | | | | | |
|  | 3 | Legislation to reduce phosphate inputs to lakes and rivers was introduced in Switzerland in 1986. This would have had the opposite effect to the observed increase in pH: less phosphate → less algal growth → more CO_2_ →lower pH. It is more likely that the higher pH reflects increased algal growth due to higher temperatures, more sunshine and a higher CO_2_ concentration or increased weathering. | | | | | |
| **ah** | **North Sea phytoplankton biomass** | | *51°N-61°N, 3°W-10°E* | | 1946-2011 | **Colour categories** | **1951, 1985** |
|  | 1 | Sir Alister Hardy Foundation for Ocean Science (SAHFOS). | | | |  |  |
|  | 2 | SAHFOS, Plymouth, UK | | | |  |  |
|  | 3 | Unit details: Four colour categories calibrated by acetone extracts and fluorescence | | | | | |
| **ai** | **North Sea temperature** | | *50°N-61°N and 3°W-9°E* | | 1950-2011 | **°C** | **1987, 2001** |
|  | 1 | Simon A. Good |  | |  |  |  |
|  | 2 | UK Met Office Hadley Centre, Exeter; EN3: quality controlled subsurface ocean temperature and salinity dataset. See: http://www.metoffice.gov.uk/hadobs/en3/ | | | | | |
| **aj** | **North Sea 50 m depth salinity** | | *58.13°N, 9.18°E* | | 1965-2007 | **­-** | **1988** |
|  | 1 | Else Juul Green, http://ocean.ices.dk/HydChem/HydChem.aspx?plot=yes | | | | | |
|  | 2 | International Council for the Exploration of the Seas, Copenhagen, Denmark | | | | | |
|  | 3 | The sampling location is equivalent to the Norwegian station Z220, which is 20 miles from the Norwegian coast. Measurements have been taken approximately once a month. | | | | | |
| **ak** | **Japan Sea temperature at 50 m depth** | | *33-38°N, 130-136°E* | | 1964-2008 | **°C** | **1987** |
|  | 1 | Yongjun Tian from the Japan Sea National Fisheries Research Institute. | | | | | |
|  | 2 | Japan Sea National Fisheries Research Institute, Niigata, Japan. | | | | | |
|  | 3 | Monthly measurements taken in the Japan Sea. to cover the path of the Tsushima Current between Wakasa Bay in Kyoto Prefecture and Yamaguchi Prefecture, Japan and averaged for the area within 33-38°N, 130-136°E. | | | | | |
| **al** | **North Pacific Kuroshio current flow** | | *across 137°E between 3-34°N* | | 1972-2011 | **1 Sv =**  **10^6^ m^3^ s^-1^** | **1987** |
|  | 1 | Yongjun Tian from theJapan Meteorological Agency (JMA), Tokyo, Japan. | | | |  |  |
|  | 2 | Japan Sea National Fisheries Research Institute, Niigata, Japan. | | | | | |
|  | 3 | Current flow estimated from geostrophic calculations based on temperature and salinity profiles taken twice a year (summer = Jul-Sep and winter = Jan-Mar) on a standard north to south section during research cruises of the Japan Meteorological Agency (JMA). | | | | | |
| **am** | **Japan Sea deep living fish (eggs)** | | *between 34.45°N-41.17°N and* | | 1981-2005 | **No. of eggs per m^2^** | **1988** |
|  | 1 | Yongjun Tian from theJapan Meteorological Agency (JMA), Tokyo, Japan. | | | |  |  |
|  | 2 | Japan Sea National Fisheries Research Institute, Niigata, Japan. | | | | | |
|  | 3 | Sampling stations were located within approximately 185 km of the coast of Japan. This abundant mesopelagic (1000-100 m deep) species normally swims between 150 to 250 m during the daytime and migrates to shallower depths during the night. | | | | | |
| **an** | **Japan Sea tuna catch** | | *34°N-41.5°N, 131°E-141°E* | | 1964-2004 | **10^3^ tons** | **1991** |
|  | 1 | Yongjun Tian from theJapan Meteorological Agency (JMA), Tokyo, Japan. | | | |  |  |
|  | 2 | Japan Sea National Fisheries Research Institute, Niigata, Japan. | | | | | |
|  | 3 | Mostly comprising warm-water bluefin, albacore, and yellowfin tuna. | | | | | |
| **ao** | **Germany lake algal spring bloom** | | *13.65°E, 52.43°N* | | 1980-2010 | **Calendar week** | **1987** |
|  | 1 | Rita Adrian | | | |  |  |
|  | 2 | Leibniz- Institute of Freshwater Ecology and Inland Fisheries, Berlin, Germany. | | | | | |
|  | 3 | The timing refers to the calendar week of the year when maximum total phytoplankton biomass developed after ice-off. | | | | | |
| **ap** | **Swiss groundwater temperature** | | *Pump-stations: Kiesen 46.80°N, 7.57°E;Neuhausen 47.68°N, 8.61°E* | | 1970-2005 | **°C** | **1987** |
|  | 1 | Simon Figura |  |  |  |  |  |
|  | 2 | Energie Wasser Bern and Städtische Werke Schaffhausen und Neuhausen am Rheinfall | | | | | |
| **aq** | **Swiss Lake Zürich temperature** | | *47.37°N-47.20°N, 8.53°E-8.82°E* | | 1946-2005 | **°C** | **1987** |
|  | 1 | Ryan P. North. | | | |  |  |
|  | 2 | Oliver Köster from Wasserversorgung der Stadt Zürich | | | | | |
| **ar** | **SH Annular Mode Index (SAM)** | | *40°S and 65°S* | | 1957-2012 | **­-** | **1992, 1996** |
|  | 1 | http://www.nerc-bas.ac.uk/icd/gjma/sam.html | | | |  |  |
|  | 2 | British Antarctic Survey, Cambridge, UK | | | | | |
|  | 3 | The index used here is derived from a proxy zonal mean sea level pressure for 40°S and 65°S calculated from twelve meteorological stations that approximate to each of these latitudes, anomalies relative to 1971-2000. | | | | | |

**Table S4**. Coordinates for six regions of tropical hurricane/storms in Fig. 6. The six regions (A-F, outlined in Fig. 6) are averaged to give a ‘global’ total for tropical hurricane/storm days.

| Ocean region | Minimum  latitude | Maximum  latitude | Minimum  longitude | Maximum  longitude |
| --- | --- | --- | --- | --- |
| A Eastern North Pacific | 5°N | 20°N | 90°W | 120°W |
| B North Atlantic | 5°N | 25°N | 20°W | 90°W |
| C North Indian | 5°N | 20°N | 55°E | 90°E |
| D South Indian | 5°S | 20°S | 50°E | 115°E |
| E Western North Pacific | 5°N | 20°N | 120°E | 180°E |
| F Southwest Pacific | 5°S | 20°S | 155°E | 180°E |

**Table S5.** Shift year of the time series from Fig. 2 included in the regions of Fig. 6.

| **EUROPE INSET** | |  |
| --- | --- | --- |
| 2b | Switzerland Payerne ~5 km tropospheric air temperature, atmosphere | 1988 |
| 2t | Baltic Sea sea-ice extent, cryosphere | 1987 |
| 2ac | UK sand martin arrival date, terrestrial biosphere | 1988 |
| 2ad | Germany grape vine ripening date, terrestrial biosphere | 1987 |
| 2ae | Baltic river Daugava winter flow, terrestrial hydrosphere | 1987 |
| 2af | Switzerland river temperature, terrestrial hydrosphere | 1987 |
| 2ah | North Sea phytoplankton biomass, marine biosphere | 1985 |
| 2ai | North Sea temperature, marine hydrosphere | 1987 |
| 2ao | Germany Lake Müggelsee algal bloom spring timing, terrestrial biosphere | 1987 |
| **JAPAN INSET** | |  |
| 2ak | Japan Sea temperature at 50m depth, marine hydrosphere | 1987 |
| 2al | Western North Pacific Kuroshio current flow, marine hydrosphere | 1987 |
| 2ab | Japan Kyoto cherry blossom blooming, terrestrial biosphere | 1988 |
| **GLOBAL MAP** | |  |
| **North America** | |  |
| 2m | Alaska Point Barrow atmospheric CO_2_ concentration (Apr-Sep), atmosphere | 1985 |
| 2n | Alaska Point Barrow atmospheric CO_2_ concentration (Oct-Mar), atmosphere | 1986 |
| 2w | Western USA Wildfire duration (days), terrestrial biosphere | 1985 |
| **Asia** | |  |
| 2e | China dust storm frequency (March-May), atmosphere | 1984 |
| **Tropics** | |  |
| 2f | Global tropical hurricane/storm days (mean areas A-F), atmosphere | 1988 |
| **Antarctica** | |  |
| 2p | Western Antarctica air surface temperature Byrd station, atmosphere | 1986 |
| 2q | Western Antarctica sea-ice extent, cryosphere | 1987 |
| **GLOBAL AND HEMISPHERIC TIME SERIES** | |  |
| Arrows left to right: | |  |
| 2n | Global CO_2_ net land uptake, atmosphere | 1988 |
| 2r | Northern Hemisphere spring snow extent, cryosphere | 1987 |
| 2x | Northern Hemisphere vegetation from satellites, terrestrial biosphere | 1987 |
| 2j | Arctic combined sea and air surface temperature, atmosphere | 1987 |
| 2k | Arctic sea level pressure, atmosphere | 1987 |
| 2c | Meridional wind speed 60-75°N (360°) ~5 km above sea level, atmosphere | 1988 |
| 2d | Zonal wind speed 60-75°N (360°) ~5 km above sea level, atmosphere | 1988 |

**Additional References for Supporting Information table S2**

Aono Y, Kazui K (2008) Phenological data series of cherry tree flowering in Kyoto, Japan, and its application to reconstruction of springtime temperatures since the 9th century. International Journal of Climatology*,* **28**, 905-914.

Axell L, Lindquist K (2005) Reconstruction of Annual Maximum Ice Extent in the Baltic Sea 1660-2005. In: *Fifth Workshop on BalticSea Ice Climate.* (eds Schrum C, Schmelzer N), pp. 79-80, Bundesamt für Seeschifffahrt und Hydrographie, Hamburg, Germany.

Barichivich J, Briffa KR, Myneni R *et al.* (2013) Large-scale variations in the vegetation growing season and annual cycle of atmospheric CO_2_ at high northern latitudes from 1950 to 2011. Global Change Biology*,* **19**, 3167-3183.

Barichivich J, Briffa KR, Osborn TJ, Melvin TM, Caesar J (2012) Thermal growing season and timing of biospheric carbon uptake across the Northern Hemisphere. Global Biogeochemical Cycles*,* **26**, GB4015.

Beaulieu C, Sarmiento JL, Mikaloff Fletcher SE, Chen J, Medvigy D (2012) Identification and characterization of abrupt changes in the land uptake of carbon. Global Biogeochemical Cycles*,* **26**, GB1007.

Bock A, Sparks T, Estrella N, Menzel A (2011) Changes in the phenology and composition of wine from Franconia, Germany. Climate Research*,* **50**, 69-81.

Brocard E, Jeannet P, Begert M, Levrat G, Philipona R, Romanens G, Scherrer SC (2013) Upper air temperature trends above Switzerland 1959–2011. Journal of Geophysical Research: Atmospheres*,* **118**, 4303-4317.

Bromwich DH, Nicolas JP, Monaghan AJ, Lazzara MA, Keller LM, Weidner GA, Wilson AB (2013) Central West Antarctica among the most rapidly warming regions on Earth. Nature Geoscience*,* **6**, 139-145.

Brown RD, Robinson DA (2011) Northern Hemisphere spring snow cover variability and change over 1922-2010 including an assessment of uncertainty. The Cryosphere*,* **5**, 219–229.

Chung U, Mack L, Yun JI, Kim S-H (2011) Predicting the timing of cherry blossoms in Washington, DC and Mid-Atlantic states in response to climate change. PLoS One*,* **6**, e27439.

Compo GP, Whitaker JS, Sardeshmukh PD *et al.* (2011) The Twentieth Century Reanalysis Project. Quarterly Journal of the Royal Meteorological Society*,* **137**, 1-28.

Danielssen DS, Svendsen E, Ostrowski M (1996) Long-term hydrographic variation in the Skagerrak based on the section Torungen–Hirtshals. ICES Journal of Marine Science*,* **53**, 917–925.

Defila C, Clot B (2001) Phytophenological trends in Switzerland. International Journal of Biometeorology*,* **45**, 203-207.

Ding R, Li J, Wang S, Ren F (2005) Decadal change of the spring dust storm in northwest China and the associated atmospheric circulation. Geophysical Research Letters*,* **32**, L02808.

Figura S, Livingstone DM, Hoehn E, Kipfer R (2011) Regime shift in groundwater temperature triggered by the Arctic Oscillation. Geophysical Research Letters*,* **38**, L23401.

Fujino T, Goto T, Shimura T *et al.* (2013) Decadal variation in egg abundance of a mesopelagic fish, *Maurolicus japonicus*, in the Japan Sea during 1981-2005. Journal of Marine Science and Technology*,* **21**, 58-62.

Gerten D, Adrian R (2000) Climate-driven changes in spring plankton dynamics and the sensitivity of shallow polymictic lakes to the North Atlantic Oscillation. Limnology and Oceanography*,* **45**, 1058-1066.

Hansen J, Sato M, Ruedy R, Lo K, Lea DW, Medina-Elizade M (2006) Global temperature change. Proceedings of the National Academy of Sciences of the United States of America*,* **103**, 14288-14293.

Hari RE, Livingstone DM, Siber R, Burkhardt-Holm P, Güttinger H (2006) Consequences of climatic change for water temperature and brown trout populations in Alpine rivers and streams. Global Change Biology*,* **12**, 10-26.

Hurrell JW (1995) Decadal trends in the North Atlantic Oscillation: regional temperatures and precipitation. Science, **269**, 676-679.

Ingleby B, Huddleston M (2007) Quality control of ocean temperature and salinity profiles — Historical and real-time data. Journal of Marine Systems*,* **65**, 158-175.

Jakob A, Binderheim-Bankay E, Davis JS (2002) National long-term surveillance of Swiss rivers. Verhandlungen Internationale Vereinigung für theoretische und angewandte Limnologie*,* **28**, 1101-1106.

Japan Meteorological Agency (2006) The Medical Examination of the Sea: a Synthetic Examination (First Edition, in Japanese), pp. 196.

Jones PD, Lister DH, Osborn TJ, Harpham C, Salmon M, Morice CP (2012) Hemispheric and large-scale land-surface air temperature variations: An extensive revision and an update to 2010. Journal of Geophysical Research: Atmospheres*,* **117**, D05127.

Kennedy JJ, Rayner NA, Smith RO, Parker DE, Saunby M (2011) Reassessing biases and other uncertainties in sea surface temperature observations measured in situ since 1850: 1. Measurement and sampling uncertainties. Journal of Geophysical Research: Atmospheres*,* **116**, D14103.

Klavins M, Briede A, Rodinov V (2009) Long term changes in ice and discharge regime of rivers in the Baltic region in relation to climatic variability. Climatic Change*,* **95**, 485-498.

Lawrimore JH, Menne MJ, Gleason BE, Williams CN, Wuertz DB, Vose RS, Rennie J (2011) An overview of the Global Historical Climatology Network monthly mean temperature data set, version 3. Journal of Geophysical Research: Atmospheres*,* **116**, D19121.

Lindsay RW, Zhang J, Schweiger A, Steele M, Stern H (2009) Arctic sea ice retreat in 2007 follows thinning trend. Journal of Climate*,* **22**, 165-176.

Marshall GJ (2003) Trends in the Southern Annular Mode from observations and reanalyses. Journal of Climate*,* **16**, 4134-4143.

Marty C (2008) Regime shift of snow days in Switzerland. Geophysical Research Letters*,* **35**, L12501.

Morice CP, Kennedy J.J, Rayner NA, Jones PD (2012) Quantifying uncertainties in global and regional temperature change using an ensemble of observational estimates: The HadCRUT4 dataset. Journal of Geophysical Research: Atmospheres*,* **117**, D08101.

Myneni RB, Keeling CD, Tucker CJ, Asrar G, Nemani RR (1997) Increased plant growth in the northern high latitudes from 1981 to 1991. Nature*,* **386**, 698-702.

North RP, Livingstone DM, Hari RE, Köster O, Niederhauser P, Kipfer R (2013) The physical impact of the late 1980s climate regime shift on Swiss lakes and rivers. Inland Waters*,* **3**, 341-350.

Ogi M, Yamazaki K, Tachibana Y (2004) The summertime annular mode in the Northern Hemisphere and its linkage to the winter mode. Journal of Geophysical Research: Atmospheres*,* **109**, D20114.

Parkinson CL, Cavalieri DJ (2008) Arctic sea ice variability and trends, 1979–2006. Journal of Geophysical Research: Oceans*,* **113**, C07003.

Raitsos DE, Pradhan Y, Lavender SJ, Hoteit I, Mcquatters-Gollop A, Reid PC, Richardson AJ (2013) From silk to satellite: half a century of ocean colour anomalies in the Northeast Atlantic. Global Change Biology, **20** 2117-23.

Rayner NA, Parker DE, Horton EB *et al.* (2003) Global analyses of sea surface temperature, sea ice, and night marine air temperature since the late nineteenth century. Journal of Geophysical Research: Atmospheres*,* **108**, 4407.

Reid PC, Edwards M, Hunt HG, Warner AJ (1998) Phytoplankton change in the North Atlantic. Nature*,* **391**, 546.

Sarmiento JL, Gloor M, Gruber N *et al.* (2010) Trends and regional distributions of land and ocean carbon sinks. Biogeosciences*,* **7**, 2351- 2367.

Sigg L, Stumm W (2011) *Aquatische Chemie. Einführung in die Chemie natürlicher Gewässer.*Zürich, vdf Hochschulverlag AG an der ETH Zürich.

Sparks T, Tryjanowski P (2007) Patterns of spring arrival dates differ in two hirundines. Climate Research*,* **35**, 159-164.

Thompson DWJ, Wallace JM (1998) The Arctic Oscillation signature in the wintertime geopotential height and temperature fields. Geophysical Research Letters*,* **25**, 1297-1300.

Thoning KW, Tans PP, Komhyr WD (1989) Atmospheric carbon dioxide at Mauna Loa Observatory: 2. Analysis of the NOAA GMCC data, 1974–1985. Journal of Geophysical Research: Atmospheres*,* **94**, 8549-8565.

Tian Y, Kidokoro H, Watanabe T, Iguchi N (2008) The late 1980s regime shift in the ecosystem of Tsushima warm current in the Japan/East Sea: evidence from historical data and possible mechanisms. Progress in Oceanography*,* **77**, 127-145.

Webster PJ, Holland GJ, Curry JA, Chang H-R (2005) Changes in tropical cyclone number, duration, and intensity in a warming environment. Science, **309**, 1844-1846.

Westerling AL, Hidalgo HG, Cayan DR, Chang H-R (2006) Warming and earlier spring increase western U.S. forest wildfire activity. Science. **313**, 940-943.

Xiao D, Li J, Zhao P (2012) Four-dimensional structures and physical process of the decadal abrupt changes of the northern extratropical ocean–atmosphere system in the 1980s. International Journal of Climatology*,* **32**, 983-994.
